# Optimizing Image Classification: Automated Deep Learning Architecture Crafting with Network and Learning Hyperparameter Tuning

**DOI:** 10.3390/biomimetics8070525

**Published:** 2023-11-04

**Authors:** Koon Meng Ang, Wei Hong Lim, Sew Sun Tiang, Abhishek Sharma, Marwa M. Eid, Sayed M. Tawfeek, Doaa Sami Khafaga, Amal H. Alharbi, Abdelaziz A. Abdelhamid

**Affiliations:** 1 Faculty of Engineering, Technology and Built Environment, UCSI University, Kuala Lumpur 56000, Malaysia; 1001436889@ucsiuniversity.edu.my (K.M.A.); tiangss@ucsiuniversity.edu.my (S.S.T.); 2 Department of Computer Science and Engineering, Graphic Era Deemed to be University, Dehradun 248002, India; abhishek15491@gmail.com; 3 Delta Higher Institute for Engineering and Technology, Mansoura 35511, Egypt; mmm@ieee.org; 4 Faculty of Artificial Intelligence, Delta University for Science and Technology, Mansoura 35111, Egypt; 5 Department of Computer Sciences, College of Computer and Information Sciences, Princess Nourah bint Abdulrahman University, P.O. Box 84428, Riyadh 11671, Saudi Arabia; dskhafga@pnu.edu.sa (D.S.K.); ahalharbi@pnu.edu.sa (A.H.A.); 6 Department of Computer Science, Faculty of Computer and Information Sciences, Ain Shams University, Cairo 11566, Egypt; abdelaziz@su.edu.sa; 7 Department of Computer Science, College of Computing and Information Technology, Shaqra University, Sahqra 11961, Saudi Arabia

**Keywords:** automatic network design, deep learning architecture, hyperparameter optimization, image classification, teaching–learning-based optimization

## Abstract

This study introduces ETLBOCBL-CNN, an automated approach for optimizing convolutional neural network (CNN) architectures to address classification tasks of varying complexities. ETLBOCBL-CNN employs an effective encoding scheme to optimize network and learning hyperparameters, enabling the discovery of innovative CNN structures. To enhance the search process, it incorporates a competency-based learning concept inspired by mixed-ability classrooms during the teacher phase. This categorizes learners into competency-based groups, guiding each learner’s search process by utilizing the knowledge of the predominant peers, the teacher solution, and the population mean. This approach fosters diversity within the population and promotes the discovery of innovative network architectures. During the learner phase, ETLBOCBL-CNN integrates a stochastic peer interaction scheme that encourages collaborative learning among learners, enhancing the optimization of CNN architectures. To preserve valuable network information and promote long-term population quality improvement, ETLBOCBL-CNN introduces a tri-criterion selection scheme that considers fitness, diversity, and learners’ improvement rates. The performance of ETLBOCBL-CNN is evaluated on nine different image datasets and compared to state-of-the-art methods. Notably, ELTLBOCBL-CNN achieves outstanding accuracies on various datasets, including MNIST (99.72%), MNIST-RD (96.67%), MNIST-RB (98.28%), MNIST-BI (97.22%), MNST-RD + BI (83.45%), Rectangles (99.99%), Rectangles-I (97.41%), Convex (98.35%), and MNIST-Fashion (93.70%). These results highlight the remarkable classification accuracy of ETLBOCBL-CNN, underscoring its potential for advancing smart device infrastructure development.

## 1. Introduction

The rapid rise of machine learning and deep learning methods has captured the attention of researchers and data science practitioners in diverse industries. These data-driven approaches have proven highly effective in handling large datasets, leveraging their computational power to extract valuable insights. Among artificial neural networks (ANNs), including feedforward neural networks (FNNs), recurrent neural networks (RNNs), and convolutional neural networks (CNNs), CNNs have gained significant popularity due to their exceptional real-world performance. 

CNNs efficiently process input images, eliminating the need for manual data preprocessing by incorporating a feature extraction module and a classifier. The feature extraction module, consisting of convolution and pooling layers, automatically captures meaningful information from raw input data during rigorous network training. These extracted features are then used by the classifier, composed of fully connected layers, ensuring consistent and reliable performance for specific tasks. Furthermore, CNNs offer flexibility and scalability, making them an attractive choice for handling complex and diverse datasets. As a result, they have found successful applications in various fields, such as action recognition [1,2], medical disease diagnosis [3,4], crack detection [5], and object classification [6]. Other notable computer vision applications of CNNs and other deep learning methods include measuring crack widths in the construction industry [7,8] and counting fruits in agriculture [9].

While CNNs have demonstrated remarkable performance and are widely acknowledged as the gold standard for deep learning tasks, designing an efficient CNN architecture capable of handling diverse datasets with varying complexity remains a challenging undertaking, often demanding specialized expertise [10]. The CNN architecture design process entails identifying the most effective combinations of network elements, encompassing both architectures and hyperparameters. Typically, the performance of CNNs hinges on two critical factors: trainable parameters and architecture [11]. Gradient descent algorithms have demonstrated their efficacy in optimizing the trainable parameters (i.e., weights and biases). However, explicit functions for optimizing the ideal CNN architecture required to achieve promising outcomes on specific datasets remain elusive [11].

Pretrained models like ResNet, GoogLeNet, MobileNet, AlexNet, and VGGNet have gained popularity for their exceptional performance in deep learning tasks. Despite differences in network architecture, including basic unit blocks, layer count, and interconnections, these pretrained models are manually crafted by human experts relying on their domain knowledge [12]. In essence, these pretrained CNN architectures are hand-crafted and lack the ability to autonomously learn the optimal configurations for competitive dataset solutions. The manual design process involves extensive trial-and-error experimentation, resulting in inefficiencies and time consumption. Moreover, these manually designed networks often struggle to adapt to various datasets, limiting their generalization capabilities [11].

As a result, there is an increasing need for automated methods capable of crafting CNN architectures based on the dataset’s characteristics, reducing the reliance on human expertise. Creating an automated approach to CNN architecture design that can adjust to diverse datasets is vital for enhancing CNN efficiency and effectiveness. These automated methods have the potential to tailor architectures to specific task requirements, ultimately enhancing the generalization capabilities of CNNs.

### 1.1. Recent Advances in Automated Network Architecture Design

In recent years, researchers and data scientists have explored various approaches to mitigate the challenges associated with manually crafting CNN architectures. This manual process can be laborious and time-consuming. Advancements in automated network architecture design have given rise to four primary approaches: reinforcement learning (RL)-based [13,14,15], gradient descent (GD)-based [16], Bayesian optimization (BO)-based [17,18,19], and metaheuristic search algorithm (MSA)-based [20,21,22] methods.

Progressive Neural Architecture Search (PNAS) [13] introduced an RL-based approach that showed superior classification performance on CIFAR-10 and ImageNet datasets. Utilizing a sequential model-based optimization (SMBO) strategy, PNAS evolved network structures from simple to complex models, learning from promising models and exploring feasible regions. PNAS achieved an eight-fold computational efficiency improvement over its predecessor, NAS [23], in image classification tasks. Efficient Neural Network Architecture Search (ENAS) [14], another RL-based approach, enhanced PNAS and NAS by conducting searches in a cell-based search space. ENAS shared parameter information among child models to discover better network architectures. ENAS delivered a thousand-fold improvement in computational efficiency compared to standard neural architecture design methods while achieving superior classification accuracy on CIFAR-10 datasets. However, RL-based approaches often require substantial computational resources for effective deep learning tasks.

A GD-based method, introduced as gradient descent NAS [16], outperformed traditional methods and long short-term memory (LSTM) in estimating remaining useful life. Importantly, this method consumed only one-third of the computational power required by RL-based approaches, highlighting its superior efficiency. Differentiable Architecture Search (DARTS) [24], another popular GD-based method, also exhibited better efficiency compared to RL-based methods. However, it encountered issues such as unstable architecture searches due to random channel selection and inefficient memory usage during network training. Various mechanisms, including cyclic feedback [25], channel attention [26,27], and self-distillation [28], were introduced to address the limitations of the original DARTS. Nonetheless, most GD-based methods often depend on domain experts to enhance their performance in designing effective CNN models.

BO is employed to explore neural architecture solution spaces through sequential search. NASBOT [17] is a Gaussian process-based BO framework designed for neural architecture search, utilizing a metric called OTMANN to measure network similarity. BayesNAS [18] addressed the limitations of one-shot NAS methods by modeling architecture parameters with hierarchical automatic relevance determination. While it reduced search time for obtaining candidate architectures, it faced computational inefficiency due to the need to cache all feature maps for Hessian computation. In a different study [19], BO was utilized to optimize RNN architectures more efficiently, utilizing three fixed-length encoding schemes and a mean absolute random sampling method. Bayesian methods include Bayesian networks offer an interpretable approach to machine learning and optimization grounded in probability theory [29]. Additionally, a Bayesian framework was applied to infer the structural composition of biological tendons, coupled with a finite element model [30].

MSAs offer an alternative solution for automated neural architecture design with minimal reliance on human domain expertise. MSAs are population-based algorithms that utilize search operators inspired by natural phenomena or organism behaviors to iteratively seek optimal solutions in optimization problems. Over the years, various MSAs have been proposed, including the artificial bee colony (ABC), the black hole algorithm (BHA), teaching–learning-based optimization (TLBO), and the whale optimization algorithm (WOA). These algorithms are characterized by their simplicity, independence from gradient information, and good global search capabilities. Consequently, MSAs have found widespread use in addressing a variety of real-world problems [31,32,33,34].

### 1.2. Existing Challenges of MSA-Based Automated Network Architecture Design

MSA-based methods hold the potential for automating the design of optimal CNN architectures for specific datasets. Nevertheless, several fundamental challenges must be addressed. One key issue is the lack of prior knowledge about optimal CNN architectures, as they involve various network and learning hyperparameters, including network depth, layer types, kernel size, filter numbers, pooling types, optimizer type, learning rate, initializer, and L2 regularization. To overcome this, an appropriate encoding strategy is crucial, enabling the representation of potential CNN architectures with variable network lengths without incurring unnecessary complexity. It is also vital for the solution encoding scheme used by MSA-based methods to ensure the validity of constructed networks while maintaining the ability to discover novel architectures. Moreover, the utilization of population-based MSA approaches presents a challenge due to the extensive computational time and resources required for evaluating candidate solutions. Therefore, there is a need for a fitness evaluation process that improves computational efficiency, making MSAs more practical for optimizing CNN network and learning hyperparameters.

Despite the introduction of various MSAs inspired by different sources in recent years, classical MSAs like particle swarm optimization (PSO), differential evolution (DE), and the genetic algorithm (GA) have predominated in optimizing CNN architectures. However, in light of the “No Free Lunch Theorem” [35], it is crucial to explore the capabilities of emerging MSAs, including ABC, BHA, TLBO, and WOA, for addressing complex real-world optimization problems like automated CNN architecture design. This exploration is essential for advancing the field and pushing the boundaries of optimization research in the context of deep learning.

TLBO has recently emerged as a promising automated approach for designing CNN architectures based on given datasets [36]. However, the search mechanisms primarily draw inspiration from the original TLBO, which has its limitations. A significant concern is that, during the teacher phase, all learners rely solely on the guidance provided by the teacher and population mean. This approach overlooks potentially valuable information possessed by other learners. The original TLBO demonstrates rapid convergence but is susceptible to premature convergence if both the teacher and population mean are entrapped in local optima during the early stage of optimization. Addressing this limitation would involve incorporating search information from better-performing learners, enabling more tailored guidance for each learner.

Furthermore, the learner phase of the original TLBO lacks an effective collaborative learning mechanism, as it restricts each learner to interacting with only one randomly selected peer. Allowing learners to interact with multiple peers or retain knowledge from previous learning can enhance the learning process’s efficiency. Lastly, the original TLBO employs a greedy selection scheme based solely on fitness criteria to determine learner survival in the next generation. Despite its simplicity, this scheme may overlook potentially superior learners with temporarily lower fitness values but the long-term potential to enhance overall population quality. These limitations compromise the delicate balance between exploitation and exploration, ultimately impacting TLBO’s performance in solving complex tasks, such as optimizing network and learning hyperparameters for CNNs.

### 1.3. Research Objectives and Contributions of Current Works

This study presents an enhanced variant, enhanced TLBO with competency-based learning (ETLBOCBL), which is designed to autonomously search for optimal CNN architectures, delivering competitive accuracy in image classification tasks of varying complexity without human intervention. ETLBOCBL incorporates several modifications in the teacher phase, learner phase, and selection scheme to strike a better balance between exploration and exploitation, thus improving its effectiveness in discovering novel CNN architectures. The primary contributions of this study are as follows:

- ETLBOCBL-CNN is an automated network design approach for discovering optimal CNN architectures for specific classification tasks. It harnesses ETLBOCBL’s optimization capability to identify the best combinations of network hyperparameters (e.g., network depth, layer types, kernel size, filter numbers, pooling size, pooling stride, and neuron numbers) and learning hyperparameters (e.g., optimizer type, learning rate, initializer type, and L2-regularizer) without human intervention.
- ETLBOCBL-CNN incorporates an efficient solution encoding scheme, enabling the search for CNN architectures of varying lengths for diverse datasets while ensuring model validity and promoting the discovery of novel architectures. Moreover, it employs an efficient fitness evaluation process for practicality.
- In ETLBOCBL-CNN, a competency-based learning concept is integrated into the modified teacher phase to encourage exploration and prevent convergence towards local optima. Learners are grouped based on their competency levels, with the more proficient learners collaborating with the teacher solution and population mean to provide more effective guidance to those with lower competence, promoting the discovery of promising CNN architectures.
- To enhance ETLBOCBL-CNN’s robustness against premature convergence, a stochastic peer interaction scheme is introduced in the modified learner phase. This scheme emulates collaborative learning dynamics observed in a classroom, enabling each learner to effectively use available information during the search process by engaging in knowledge sharing and retention with one or multiple peer learners.
- In ETLBOCBL-CNN, a tri-criterion selection scheme is introduced as an enhanced alternative to the conventional greedy selection method. This new selection scheme determines learners’ survival in subsequent iterations by considering their fitness, diversity, and improvement rates. The proposed scheme preserves valuable network information and contributes to long-term population quality improvement by favoring learners with relatively good diversity and commendable fitness improvement, even if their current fitness levels are temporarily lower.
- Extensive simulation studies are performed on image datasets with varying complexity to assess the effectiveness and feasibility of ETLBOCBL-CNN in autonomously discovering optimal CNN architectures. The findings reveal that ETLBOCBL-CNN produces superior CNN architectures, achieving excellent classification performance with reduced complexity compared to state-of-the-art methods on most datasets.

### 1.4. Paper Outline

The subsequent sections of this paper are structured as follows: Section 2 reviews related literature. Section 3 details the workflow of ETLBOCBL-CNN for automating optimal CNN architecture design. Section 4 presents simulation settings and compares ETLBOCBL-CNN results with other approaches. Finally, Section 5 provides a brief summary of conclusions and outlines potential avenues for future research.

## 2. Related Works

### 2.1. Original TLBO

TLBO was originally developed to address complex engineering design optimization tasks by modeling the knowledge acquisition process in classrooms [37]. In its initialization stage, TLBO randomly generates a group of *N* learners with *D*-dimensional size. Each learner $X_{n}$, identified by its learner index *n* and decision variable index *d*, is associated with a position vector $X_{n}.Pos=\left[X_{n}.Pos_{1},\ldots ,X_{n}.Pos_{1},\ldots ,X_{d}.Pos_{D}\right]$, which signifies a potential solution for the given problem. The learner’s knowledge level is indicated by its fitness value, $X_{n}.Fit$.

TLBO comprises two phases, each employing different learning mechanisms to enhance learners’ knowledge levels. In the teacher phase, the *n*-th learner obtains the latest knowledge by comparing the most knowledgeable teacher ($X^{Teacher}$) with the population mean (X.Mean) that represents the average knowledge level of the population. Specifically, X.Mean is calculated by averaging the position vectors of all population members.

(1) $$X.Mean=\frac{1}{N}\overset{N}{\underset{n=1}{\sum }}X_{n}.Pos$$

Each *n*-th learner calculates a new position, $X_{n}^{New}.Pos$, using Equation (2), which involves a randomly generated number $r_{1}\in \left[0,1\right]$ from a uniform distribution and a teacher factor, $F^{T}\in \left\{0,1\right\}$, influencing mainstream knowledge during the knowledge acquisition process.

(2) $$X_{n}^{New}.Pos=X_{n}.Pos+r_{1}\left(X^{Teacher}.Pos-F^{T}X.Mean\right)$$

In the learner phase, each learner, denoted as $X_{n}$, can enhance their knowledge through interactions with a randomly selected peer, $X_{m}$, where m≠n. In minimization problems, learners with smaller fitness values ($X_{n}.Fit$ and $X_{m}.Fit$) possess higher knowledge levels. The new position, $X_{n}^{New}.Pos$, is determined using Equation (3) and a randomly generated number $r_{2}\in \left[0,1\right]$. Equation (3) illustrates that each learner can either move away from a peer with inferior fitness to promote exploration or move closer to a peer with superior fitness to encourage exploitation.

(3) $$X_{n}^{New}.Pos=\{\begin{array}{c} X_{n}.Pos+r_{2}\left(X_{n}.Pos-X_{m}.Pos\right),\;\;\;\;\;\;\mathrm{if}\;X_{n}.Fit<X_{m}.Fit\;\\ X_{n}.Pos+r_{2}\left(X_{m}.Pos-X_{n}.Pos\right),\;\;\;\;\;\;\;\;\;\;\;\;\;\;\;\;\;\;\;\;\;\;\;\;\;\;\;\;\mathrm{otherwise}\; \end{array}$$

**Figure 1:**
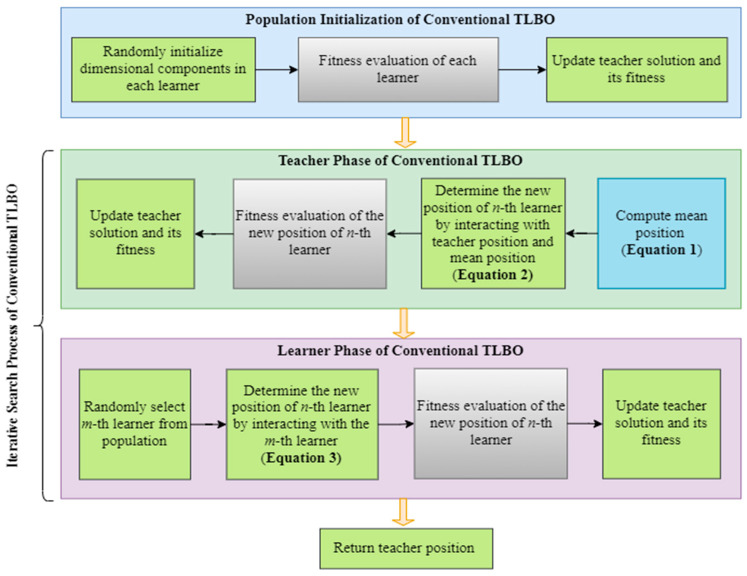
The workflow of the original TLBO.

At the end of either the teacher or learner phase, the fitness value corresponding to each *n*-th learner’s new position, $X_{n}^{New}.Fit$, is compared with their current value, $X_{n}.Fit$. If $X_{n}^{New}.Fit<X_{n}.Fit$, the updated position, $X_{n}^{New}$, replaces the current position $X_{n}$. The learning processes for each learner in both phases of TLBO iterate until the termination criteria are met, and $X^{Teacher}$ is returned to solve the given problem. The workflow of the original TLBO is depicted in the block diagram presented in Figure 1.

### 2.2. CNN

**Figure 2:**
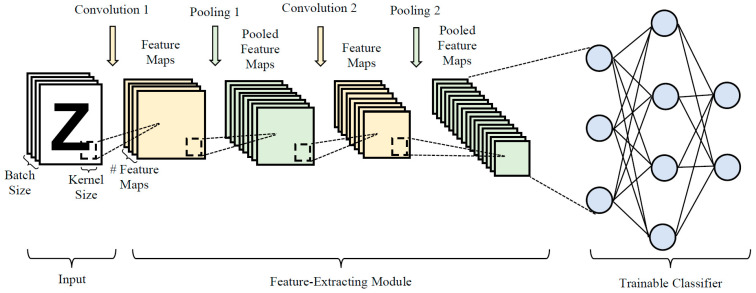
Typical architecture of sequential CNN.

CNN architecture has revolutionized deep learning research by seamlessly integrating a feature extraction module with a classification module. This innovative approach efficiently extracts crucial information from raw input data, which is then passed to the classification module for further analysis. This enhances overall efficiency and reduces potential errors associated with manual preprocessing methods. Figure 2 provides a visual representation of a typical sequential CNN architecture, featuring a feature extraction module with two convolutional blocks and two pooling blocks, alongside a classification module consisting of three fully connected blocks. Each functional block in a CNN has specific hyperparameters vital for effective network construction and training. For example, convolutional blocks have hyperparameters like kernel size and filter number, while pooling blocks encompass hyperparameters like stride size, pooling size, and pooling type. Similarly, the performance of the classification module depends on hyperparameters such as the number of fully connected blocks and the associated neuron numbers.

Two types of convolution processes are commonly observed in CNNs, i.e., SAME and VALID convolutions. SAME convolution employs zero padding to ensure that resulting feature maps have the same size as the input data, while VALID convolution produces smaller feature maps without padding. In each convolutional block, filters with predefined dimensions generate feature maps from the input data. During convolution, the filter moves horizontally with a specified stride width and, upon reaching the rightmost position, moves down with a stride height, continuing the sliding process from left to right to create a complete feature map. Feature map elements are computed by summing the products of filter elements and the corresponding input data elements captured by the filter. In addition to hyperparameters such as filter size, number, stride size, feature map number, and convolution type, connection weights within the filters are adjusted as trainable parameters during training.

Pooling is a vital component in CNNs to facilitate local translation invariance. Two common pooling techniques are average pooling, which computes the mean values of elements captured by a kernel to create down-sampled feature maps, and maximum pooling, which identifies the largest values among the captured elements. During pooling, a predefined kernel is applied to the input data, generating down-sampled feature maps by moving the kernel from the top left to the bottom right according to specified stride height and width. Pooling blocks lack trainable parameters such as connection weights, with the relevant hyperparameters involving pooling type, kernel size, and stride size.

The main objective of CNN training is to minimize the errors between predicted and actual outputs in the datasets by optimizing the trainable parameters through backpropagation and gradient descent, thereby reducing cross-entropy loss. However, training a CNN from scratch can be time-consuming due to the large number of trainable parameters involved. Moreover, traditional CNN architecture design methods relying on trial-and-error approaches can be inefficient and require significant expertise. To address these challenges, automatic network design methods offer a promising alternative to enhance efficiency in developing optimal CNN architectures for specific deep learning tasks with minimal human intervention. By automating the design process, these methods allow the model to swiftly explore and identify the most suitable network architecture and learning hyperparameters that meet performance criteria, freeing researchers to focus on other critical aspects of deep learning research.

### 2.3. Existing MSA-Based Network Architecture Design Methods

Backpropagation was initially employed in ANNs to train connection weights between neurons. However, this method often struggled in complex fitness landscapes, becoming stuck in local optima. To address this, researchers turned to MSAs for training ANNs with fixed network structures [38,39]. MSAs offered remarkable exploratory search capabilities, enabling them to find global optima in challenging ANN training problems independently of gradient information. For example, simulation studies in [39] demonstrated that GA-trained network architectures produced lower errors (0.207) compared to backpropagation (0.675) when solving eight test cases. While MSAs excelled in search accuracy, they were found to require longer computational times for larger networks [40]. In response to the limitations of backpropagation, Topology and Weight Evolving Artificial Neural Networks (TWEANNs) were introduced. These neuroevolutionary approaches not only trained connection weights but also simultaneously constructed optimal network structures. Inspired by GA, Neuroevolution of Augmenting Topologies (NEAT) [41] aimed to evolve ANNs from simpler to more complex structures, incorporating a speciation mechanism to preserve solution diversity during the network’s evolution. NEAT was validated on pole balancing tasks and was 25 times faster than cellular encoding and 5 times faster than enforced subpopulation methods. However, NEAT faced high computational costs when evolving networks with high-dimensional sizes due to the use of a direct encoding scheme. To address this, Evolutionary Acquisition of Neural Topologies (EANT) [42] proposed a two-layer optimization approach. The first layer emphasized exploration through mutation strategies for network structure evolution, while the second layer promoted exploitation through evolution strategies to identify optimal network weights. During performance validation with the simulated visual servoing task, EANT consistently outperformed NEAT and required less parameter tuning. Hypercube-Based NeuroEvolution of Augmenting Topologies (HyperNEAT) [43] emerged as an improvement over NEAT, mitigating the drawbacks of its direct encoding scheme. HyperNEAT introduced an indirect encoding scheme called Connective Compositional Pattern Producing Network (CPPN), significantly enhancing the computational efficiency required for network construction with millions of connections. HyperNEAT’s performance was evaluated through visual discrimination and food gathering tasks at varying resolutions, demonstrating its ability to discover repeating motifs in neural connectivity.

In recent years, there has been a growing trend in using MSAs to optimize complex neural network structures and parameters. One noteworthy algorithm is ABC, which draws inspiration from the foraging behavior of bee colonies and has been proposed for discovering optimal CNN architectures. For example, distributed ABC [44] was introduced to initialize the pretrained connection weights of a CNN model, with the aim of minimizing image classification errors. These pretrained connection weights were subsequently refined using the gradient descent algorithm. Moreover, different random seeds were employed to generate initial solutions within each subgroup for solution diversity preservation. The CNN optimized by distributed ABC achieved higher accuracy (97.67%) on the MNIST dataset compared to SA (96.23%), GA (96.78%), PSO (97.14%), and BA (97.23%). Importantly, the distributed ABC approach maintains computational efficiency when handling large datasets through its distributed strategy. In [45], ABC was applied to perform neuroevolution on CNNs by optimizing their architecture and training hyperparameters. A direct encoding scheme represented network information for constructing and evaluating networks, including details like the number and types of layers, kernel size, pooling size, connectivity pattern, neuron count, weight regularization, dropout rate, batch size, and learning rules. Despite demonstrating a promising ability to achieve a low error rate of 0.62% on the MNIST dataset, it was observed that ABC tends to require more time to produce reliable results. ABC was utilized to optimize the hyperparameters of CNN structures for human action recognition applications [46]. A direct encoding scheme was employed to represent over six training hyperparameters, including maximum epochs, minibatch size, initial learning rate, L2 regularization, shuffle, and momentum. This method successfully solved the sign language digit and Thomas Moeslund’s gesture recognition datasets with accuracy levels of 98.40% and 98.09%, respectively.

WOA is a promising MSA inspired by the hunting behavior of humpback whales and has demonstrated significant potential in optimizing CNN models. Notably, Dixit et al. [47] successfully applied WOA to optimize CNN hyperparameters for texture recognition tasks, including the number of filters, kernel size, weights, and biases. Extensive simulation studies revealed that this method achieved promising accuracies on datasets like Kylberg [48] (99.71%), Brodatz [49] (97.43%), and Outex [50] (97.70%). In [51], WOA was combined with SGD to optimize the connection weights and biases of a deep CNN model for efficient crowd emotion recognition, covering emotions such as normal, happy, angry, moving, violence, and fighting. The resulting SGD-WOA deep CNN exhibited superior sensitivity (96.75%), specificity (99.36%), and accuracy (96.93%) in emotion recognition. During the COVID-19 pandemic, WOA was applied to optimize the training hyperparameters of the ResNet-50 model for COVID-19 diagnosis using radiography images [52]. By leveraging WOA to optimize training hyperparameters such as momentum learning, batch size, epoch, and validation frequency, the performance of ResNet-50 was significantly enhanced. Subsequently, SGD was employed to train the trainable weights and biases of the optimized ResNet-50 model. The ResNet-50 optimized by WOA outperformed other optimization schemes, including the grey wolf optimizer (GWO), PSO, GA, simulated annealing (SA) and pattern search (PS), in terms of accuracy (98.78%), sensitivity (98.37%), specificity (99.19%), precision (99.18%), and F1 score (98.37%) when classifying the COVID-CT scan datasets.

In [53], a novel evolutionary NAS method incorporating RepVGG nodes, referred to as EvoNAS-Rep, was introduced. Initially, an encoding strategy was devised to map fixed-length encoded individuals to deep learning structures with variable lengths. Subsequently, a GA was employed to search for optimal individuals corresponding to deep learning models. EvoNAS-Rep has demonstrated its capability by achieving accuracies of 96.35% on CIFAR-10 and 79.82% on CIFAR-100 datasets. Another GA-based method with a self-adaptive mutation scheme was proposed in [54] to tackle the CNN architecture design problem using a block-design approach. This approach exhibited improved exploration through adaptive mutation strategy adjustments during architecture optimization. Simulation studies revealed that this method can efficiently tackle CIFAR-10 and CIFAR-100 datasets, achieving error rates of 3.6% and 20.2%, respectively. Furthermore, an efficient evolutionary NAS approach featuring a modular inheritable crossover operator and mutation operator was presented in [55]. The specially designed crossover operator ensured that modular information from parent architectures could be inherited by offspring architectures, thus accelerating algorithm convergence. This method reported impressive results, solving the CIFAR-10 and CIFAR-100 datasets with error rates of 2.62% and 18.46%, respectively. In [56], an efficient evolutionary NAS method was proposed, evolving CNN architectures based on a multi-branch and batch-free normalization transformer backbone for image classification tasks. It introduced a flexible encoding strategy that adaptively evolved CNN configurations with varying network depths. Both crossover and mutation operators were incorporated to strike a balance between exploration and exploitation. Simulation studies demonstrated that this evolutionary NAS method achieved accuracies of 97.24% on CIFAR-10 and 80.06% on CIFAR-100 datasets. 

In addition to the previously mentioned MSAs, various optimization algorithms inspired by different natural principles have been effectively applied to address optimization problems associated with CNNs. In a study conducted by [57], the black hole algorithm (BHA) was harnessed to seek the optimal connection weights and biases of the classifier module within a CNN model. Notably, each individual solution employed a direct encoding strategy to represent the classifier module’s weights and biases. The resulting BH-CNN achieved a classification accuracy of 96.88% when solving the MNIST dataset. Another notable application involved the development of a brain–computer interface framework using CNN and BHA to search for the optimal network structure capable of classifying perception and visual imagination based on non-invasive EEG signals [58]. BHA was used to search for the optimal numbers of convolutional layers, filter sizes, neuron numbers, and types of activation functions employed within the CNN. Experimental studies revealed that the CNN optimized by BHA could classify imagination and perception into 12 different classes with an accuracy of close to 30%. The equilibrium optimization (EO) algorithm was leveraged by EO-CNN [59] to train the connection weights and biases of a CNN model specifically designed for traffic transportation prediction tasks. EO-CNN utilized a direct encoding scheme to represent the weights and biases of CNN models. EO-CNN demonstrated competitive real-time prediction performance when handling real-time traffic data from the Twin Cities Metro. It achieved smaller mean values of root mean square error (3.869), mean squared logarithmic error (0.121), and explained variance error (0.389). A novel approach, the mutation-based Henry gas solubility optimization (MHSGO) [60], introduced a fresh perspective on optimizing hyperparameters for DenseNet-121 in plant leaf disease classification. Unlike conventional HSGO, MHSGO incorporated a mutation scheme to enhance population diversity during hyperparameter optimization, including factors such as neuron count, batch size, and learning rate. Consequently, the CNN model optimized by MHSGO outperformed other deep learning models when tested with field data featuring complex backgrounds, achieving an accuracy of 98.81%, precision of 98.60%, and recall of 98.75%. In [61], an opposition-based symbiotic organism search (OSOS) algorithm was proposed to perform hyperparameter tuning of learning rate and momentum when training a ResNet-50 model enhanced with attention residual learning mechanisms for leaf disease recognition. This optimized attention residual learning network successfully classified fifteen health conditions of eggplant, mango, guava, and citrus leaves with an accuracy of 98.20%.

## 3. Proposed ETLBOCBL-CNN

**Figure 3:**
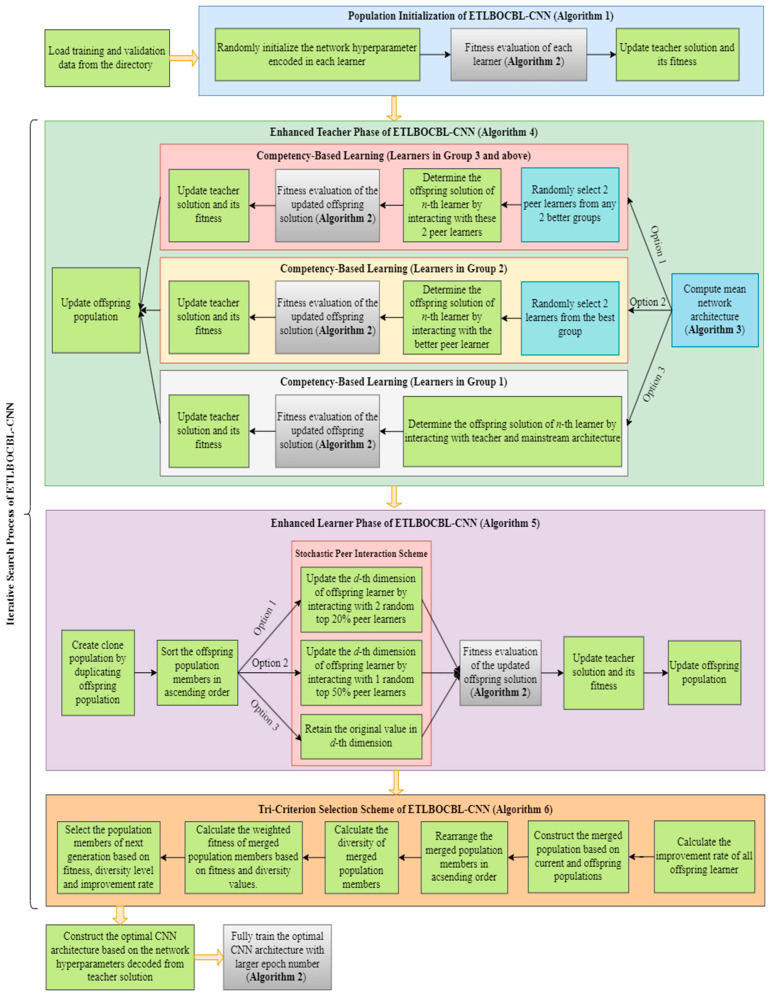
Workflow of the ETLBOCBL-CNN framework.

This study presents ETLBOCBL-CNN, an innovative approach for automatically designing efficient CNN models for image classification. This proposed method aims to construct valid and high-performing CNN architectures tailored to specific datasets with minimal human intervention. A CNN architecture is considered valid if it meets the following criteria: (a) it starts with a convolutional layer, (b) it ends with a fully connected layer, (c) it avoids inserting fully connected layers between the feature extraction module (comprising convolutional and pooling layers) to prevent overfitting and excessive trainable parameters, and (d) the number of pooling layers is limited based on the input dataset size; for example, a maximum of three pooling layers is allowed for input datasets sized at 28 × 28 × 1 [62]. Figure 3 outlines the workflow of the ETLBOCBL-CNN framework. Detailed explanations of the modifications in the teacher phase, learner phase, and selection scheme of ETLBOL-CNN will be provided in the following subsections.

### 3.1. Proposed Solution Encoding Scheme

**Figure 4:**
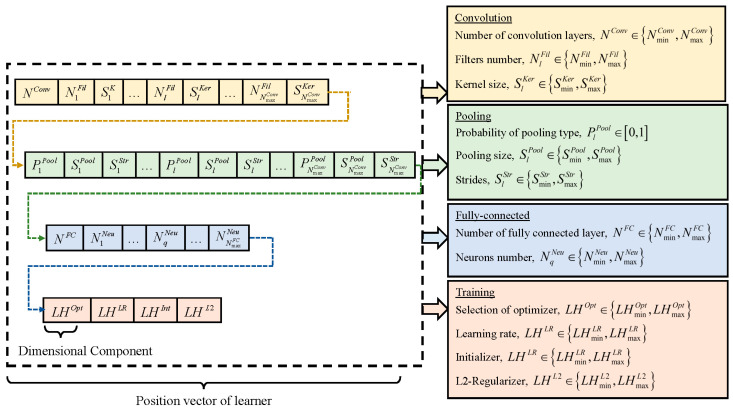
Solution encoding scheme of ETLBOCBL-CNN to represent a potential CNN.

Constructing optimal CNN models involves determining network hyperparameters, including network depth, layer types, kernel size, filter numbers, pooling size, pooling stride, and neuron number. Furthermore, selecting appropriate combinations of learning hyperparameters, such as optimizer type, learning rate, initializer type, and L2 regularization, is vital for optimizing network training and achieving competitive classification performance. In light of these considerations, ETLBOCBL-CNN introduces an efficient solution encoding scheme, which enables each learner to effectively search for optimal network and learning hyperparameters. This scheme ensures that only valid architectures are created without limiting ETLBOCBL-CNN’s ability to discover novel and effective CNN architectures for image classification. As depicted in Figure 4, each ETLBOCBL-CNN learner, denoted as the *n*-th learner, is represented by a *D*-dimensional position vector, $X_{n}.Pos$. Each *d*-th dimension, $X_{n}.{Pos}_{d}$, corresponds to specific network or learning hyperparameters required for the construction of a unique CNN architecture. These hyperparameters are divided into four main sections: convolution, pooling, fully connected, and network training.

The CNN’s convolution section is characterized by three key hyperparameters, as illustrated in Figure 4. The first hyperparameter, $N^{Conv}\in \left\{N_{min}^{Conv},N_{max}^{Conv}\right\}$, represents the number of convolution layers and is encoded in $X_{n}.{Pos}_{d}$ with d=1, where $N_{min}^{Conv}$ and $N_{max}^{Conv}$ define the minimum and maximum allowable convolution layer counts. Each convolution layer is identified by an index number, $l\in \left\{1,N_{max}^{Conv}\right\}$. For each *l*-th convolution layer, there are two additional hyperparameters: $N_{l}^{Fil}\in \left\{N_{min}^{Fil},N_{max}^{Fil}\right\}$, which signifies the number of filters, and $S_{l}^{Ker}\in \left\{S_{min}^{Ker},S_{max}^{Ker}\right\}$, which denotes the kernel size of each filter. These hyperparameters are encoded in $X_{n}.{Pos}_{d}$, with d=2l for $N_{l}^{Fil}$ and d=2l+1 for $S_{l}^{Ker}$, where $l=1,\ldots ,N_{max}^{Conv}$. It is worth noting that while all ETLBOCBL-CNN learners possess position vectors $X_{n}.Pos$ of the same dimension size *D*, they can generate CNNs with varying numbers of convolution layers by referring to the value of $N^{Conv}\in \left\{N_{min}^{Conv},N_{max}^{Conv}\right\}$ encoded in $X_{n}.{Pos}_{d}$ with d=1. If $N^{Conv}<N_{max}^{Conv}$, only the first $N^{Conv}$ values of $N_{l}^{Fil}$ and $S_{l}^{Ker}$, encoded into $X_{n}.{Pos}_{d}$ with d=2l and d=2l+1 for $l=1,\ldots ,N^{Conv}$, are utilized to construct the CNN’s convolution section. Redundant network information stored in $X_{n}.{Pos}_{d}$, with d=2l and d=2l+1 for $l=N^{Conv}+1,\ldots ,N_{max}^{Conv}$, is omitted from the network construction process.

Three hyperparameters are introduced to define the pooling section of the CNN. The first hyperparameter, $P_{l}^{Pool}\in \left[0,1\right]$, is encoded into $X_{n}.{Pos}_{d}$, with $d=2N_{max}^{Conv}+3l-1$ for $l=1,\ldots ,N_{max}^{Conv}$. It signifies the type of pooling layer connected to each *l*-th convolution layer according to the following guidelines: (a) no pooling layer is inserted when $0\leq P_{l}^{Pool}<1/3$, (b) maximum pooling is applied when $1/3\leq P_{l}^{Pool}<2/3$, and (c) average pooling is employed when $2/3\leq P_{l}^{Pool}\leq 1$. The minimum and maximum sizes of the pooling layers linked to each *l*-th convolutional layer are denoted as $S_{min}^{Pool}$ and $S_{max}^{Pool}$, while $S_{min}^{Str}$ and $S_{max}^{Str}$ represent the minimum and maximum stride sizes of the pooling layer. Two more hyperparameters, $S_{l}^{Str}\in \left\{S_{min}^{Str},S_{max}^{Str}\right\}$ and $S_{l}^{Pool}\in \left\{S_{min}^{Pool},S_{max}^{Pool}\right\}$, represent the kernel size and stride size of the pooling layer associated with the *l*-th convolution layer. These hyperparameters are encoded into $X_{n}.{Pos}_{d}$, with $d=2N_{max}^{Conv}+3l$ for $S_{l}^{Str}$ and $d=2N_{max}^{Conv}+3l+1$ for $S_{l}^{Pool}$, where $l=1,\ldots ,N_{max}^{Conv}$. Similarly, only relevant network information of $P_{l}^{Pool}$, $S_{l}^{Str}$, and $S_{l}^{Pool}$ contributes to the construction of the CNN’s pooling section. In cases where $N^{Conv}<N_{max}^{Conv}$, only the first $N^{Conv}$ values of $P_{l}^{Pool}$, $S_{l}^{Pool}$, and $S_{l}^{Str}$, encoded into $X_{n}.{Pos}_{d}$ with $d=2N_{max}^{Conv}+3l-1$, $d=2N_{max}^{Conv}+3l$, and $d=2N_{max}^{Conv}+3l+1$, respectively, for $l=1,\ldots ,N^{Conv}$, are used for generating the pooling section of the CNN. Redundant network information of $P_{l}^{Pool}$, $S_{l}^{Pool}$, and $S_{l}^{Str}$ stored in $X_{n}.{Pos}_{d}$ with $d=2N_{max}^{Conv}+3l-1$, $d=2N_{max}^{Conv}+3l$, and $d=2N_{max}^{Conv}+3l+1$, respectively, for $l=N^{Conv}+1,\ldots .,N_{max}^{Conv}$, is disregarded in network construction. It is essential to note that the network information of $S_{l}^{Pool}$ and $S_{l}^{Str}$ is excluded if the corresponding $P_{l}^{Pool}$ falls within the range of [0,1/3] since no pooling layer is introduced with the *l*-th convolution layer in this scenario.

The fully connected section of a CNN is constructed using two hyperparameters. The first hyperparameter, $N^{FC}\in \left\{N_{min}^{FC},N_{max}^{FC}\right\}$, represents the number of fully connected layers in the CNN. It is encoded into $X_{n}.{Pos}_{d}$, with $d=5N_{max}^{Conv}+2$, where $N_{min}^{FC}$ and $N_{max}^{FC}$ define the minimum and maximum allowable numbers of fully connected layers, respectively. Each fully connected layer is identified by an index number, $q\in \left\{1,N^{FC}\right\}$. The second hyperparameter, $N_{q}^{Neu}\in \left\{N_{min}^{Neu},N_{max}^{Neu}\right\}$, indicates the number of neurons in the *q*-th fully connected layer. It is encoded into $X_{n}.{Pos}_{d}$ with $d=\left(5N_{max}^{Conv}+2\right)+q$ and $q=1,\ldots ,N_{max}^{FC}$. Here, $N_{min}^{Neu}$ and $N_{max}^{Neu}$ represent the minimum and maximum numbers of neurons in a fully connected layer. Similarly to the convolution and pooling sections, only the first $N^{FC}$ values of $N_{q}^{Neu}$ encoded into $X_{n}.{Pos}_{d}$ with $d=\left(5N_{max}^{Conv}+2\right)+q$ and $q=1,\ldots ,N^{FC}$ are used in generating the fully connected section of the CNN. Any redundant information of $N_{q}^{Neu}$, stored in $X_{n}.{Pos}_{d}$ with $d=\left(5N_{max}^{Conv}+2\right)+q$ and $q=N^{FC}+1,\ldots ,\;N_{max}^{FC}$, is disregarded in network construction.

In addition to the network hyperparameters used in constructing the convolution, pooling, and fully connected sections of the CNN, four learning hyperparameters are integrated into the position vector of each ETLBOCBL-CNN learner. This integration enhances the optimization process, allowing ETLBOCBL-CNN to produce more accurate and effective CNN models. Notably, the optimization of learning hyperparameters in ETLBOCBL-CNN involves selecting the optimizer type, learning rate, initializer type, and L2-regularizer. These learning hyperparameters are represented by integer indices within the ranges of $\left\{LH_{min}^{Opt}\;,LH_{max}^{Opt}\right\}$, $\left\{LH_{min}^{LR}\;,LH_{max}^{LR}\right\}$, $\left\{LH_{min}^{Int}\;,LH_{max}^{Int}\right\}$, and $\left\{LH_{min}^{L2}\;,LH_{max}^{L2}\right\}$, respectively. To optimize the CNN training process for each ETLBOCBL-CNN learner, the selection of these learning hyperparameters is represented by the integer decision variables: $LH^{Opt}$, $LH^{LR}$, $LH^{Int}$, and $LH^{L2}$. These variables are encoded into $X_{n}.{Pos}_{d}$ with the dimension indices of $d=5N_{max}^{Conv}+N_{max}^{Neu}+3$, $d=5N_{max}^{Conv}+N_{max}^{Neu}+4$, $d=5N_{max}^{Conv}+N_{max}^{Neu}+5$, and $d=5N_{max}^{Conv}+N_{max}^{Neu}+6$, respectively.

**Figure 5:**
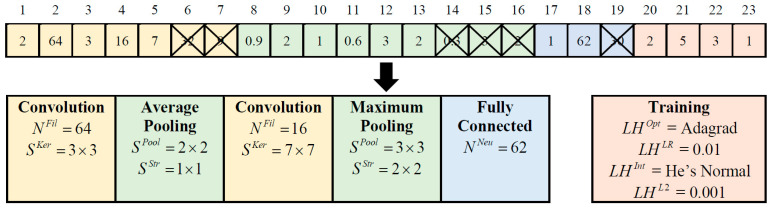
Decoding of network and learning hyperparameters for CNN construction.

The process of constructing a CNN architecture from the network and learning hyperparameters, decoded from the position vector $X_{n}.Pos$ of the *n*-th ETLBOCBL-CNN learner, is visually represented in Figure 5. Table 1 summarizes the feasible search ranges for all network and learning hyperparameters based on [62]. Meanwhile, the selection of the optimizer type, learning rate, initializer type, and L2-regularizer is based on the integer indices presented in Table 2. In this demonstration, the maximum allowable numbers of convolution and fully connected layers are defined as $N_{max}^{Conv}=3$ and $N_{max}^{FC}=2$, respectively. Consequently, the total dimension size of the position vector $X_{n}.Pos$ for each *n*-th ETLBOCBL-CNN learner is calculated as $D=5N_{max}^{Conv}+N_{max}^{FC}+6=23$. To illustrate, the values of $N^{Conv}$ and $N^{FC}$ encoded into $X_{n}.{Pos}_{d}$, with d=1 and d=17, respectively, indicate that the constructed CNN has two convolution layers, up to two pooling layers, and one fully connected layer. Specifically, the first convolution layer is generated with values $N_{1}^{Fil}=64$ and $S_{1}^{Ker}=3\times 3$, encoded into $X_{n}.{Pos}_{d}$ with d=2 and d=3, respectively. The second convolutional layer is established using values $N_{2}^{Fil}=16$ and $S_{2}^{Ker}=7\times 7$ encoded into $X_{n}.{Pos}_{d}$ with d=4 and d=5. In the pooling section of the CNN, a maximum pooling layer is inserted into the first convolution layer. This is achieved via the values of $P_{1}^{Pool}=0.9$, $S_{1}^{Pool}=2\times 2$, and $S_{1}^{Str}=1\times 1$ encoded into $X_{n}.{Pos}_{d}$ with d=8 to d=10. An averaging pooling layer is inserted into the second convolution layer based on values $P_{2}^{Pool}=0.6$, $S_{2}^{Pool}=3\times 3$, and $S_{2}^{Str}=2\times 2$ encoded into $X_{n}.{Pos}_{d}$ with d=11 to d=13. The fully connected layer of the CNN comprises 18 neurons, as indicated by the value of $N_{1}^{Neu}$ encoded into $X_{n}.{Pos}_{d}$ with d=18. The trainable parameters of the constructed CNN are initialized using He Normal and optimized using Adagrad with a learning rate of 0.01 and an L2-regularization value of 0.001. This information is revealed through the integer indices of $LH^{Opt}$, $LH^{LR}$, $LH^{Int}$, and $LH^{L2}$ encoded into $X_{n}.{Pos}_{d}$ with d=20 to d=23. It is important to note that certain network information, specifically related to the third convolutional layer, the third pooling layer, and the second fully connected layer, is excluded from the network construction.

### 3.2. Population Initialization of ETLBOCBL-CNN

Algorithm 1 outlines the procedure for initializing the ETLBOCBL-CNN population. This initialization step serves to create a diverse set of CNN architecture candidates by generating random position vectors, denoted as $X_{n}.Pos$, for *N* learners, with n=1,…,N. The total dimension size of each $X_{n}.Pos$ is computed as $D=5N_{max}^{Conv}+N_{max}^{FC}+6$. For every CNN architecture built by the *n*-th learner, the corresponding network hyperparameters ($N^{Conv}$, $N_{l}^{Fil}$, $S_{l}^{Ker}$, $P_{l}^{Pool}$, $S_{l}^{Pool}$, $S_{l}^{Str}$, $N^{FC}$, and $N_{q}^{Neu}$) and learning hyperparameters ($LH^{Opt}$, $LH^{LR}$, $LH^{Int}$, and $LH^{L2}$) are randomly generated within their predefined feasible ranges, as summarized in Table 1. These hyperparameters are then encoded into the respective $X_{n}.{Pos}_{d}$, where d=1,…,D, based on Algorithm 1.
**Algorithm 1:** Population Initialization of ETLBOCBL-CNN**Input:** *N*, $N_{min}^{Conv}$, $N_{max}^{Conv}$, $N_{min}^{Fil}$, $N_{max}^{Fil}$, $S_{min}^{Ker}$, $S_{max}^{Ker}$, $S_{min}^{Pool}$, $S_{max}^{Pool}$, $S_{min}^{Str}$, $S_{max}^{Str}$, $N_{min}^{FC}$, $N_{max}^{FC}$, $N_{min}^{Neu}$, $N_{max}^{Neu}$, $LH_{min}^{Opt}$, $LH_{min}^{LR}$, $LH_{min}^{Int},$ $LH_{min}^{L2}$, $LH_{max}^{Opt}$, $LH_{max}^{LR}$, $LH_{max}^{Int}$, $LH_{max}^{L2}$01:Compute the dimensional size as $D=5N_{max}^{Conv}+N_{max}^{FC}+6$;02:Initialize teacher solution as $X^{Teacher}.Pos\leftarrow \emptyset$ and $X^{Teacher}.Err\leftarrow \infty$;03:**for** *n* = 1 to *N* **do**04:  Initialize $X_{n}.Pos\leftarrow \emptyset$;05:  **for** *d* = 1 to *D* **do**
06:    **if** d==1 **then**07:      Assign $X_{n}.{Pos}_{d}$ with $N^{Conv}\in \left\{N_{min}^{Conv},N_{max}^{Conv}\right\}$;08:    **else if** d==2l **then**09:      Assign $X_{n}.{Pos}_{d}$ with $\;N_{l}^{Fil}\in \left\{N_{min}^{Fil},N_{max}^{Fil}\right\}$ for $\;l=1,\ldots ,N_{max}^{Conv}$;10:    **else if** d==2l+1 **then**11:      Assign $X_{n}.{Pos}_{d}$ with $S_{l}^{Ker}\in \left\{S_{min}^{Ker},S_{max}^{Ker}\right\}$ for $\;l=1,\ldots ,N_{max}^{Conv}$;12:    **else if** $d==2N_{max}^{Conv}+3l-1$ **then**13:      Assign $X_{n}.{Pos}_{d}$ with $P_{l}^{Pool}\in \left[0,1\right]$ for $\;l=1,\ldots ,N_{max}^{Conv}$;14:    **else if** $d==2N_{max}^{Conv}+3l$ **then**15:      Assign $X_{n}.{Pos}_{d}$ with $S_{l}^{Pool}\in \left\{S_{min}^{Pool},S_{max}^{Pool}\right\}$ for $\;l=1,\ldots ,N_{max}^{Conv}$;16:    **else if** $d==2N_{max}^{Conv}+3l+1$ **then**17:      Assign $X_{n}.{Pos}_{d}$ with $S_{l}^{Str}\in \left\{S_{min}^{Str},S_{max}^{Str}\right\}$ for $\;l=1,\ldots ,N_{max}^{Conv}$;18:    **else if** $d==5N_{max}^{Conv}+2$ **then**19:      Assign $X_{n}.{Pos}_{d}$ with $N^{FC}\in \left\{N_{min}^{FC},N_{max}^{FC}\right\}$;20:    **else if** $d==\left(5N_{max}^{Conv}+2\right)+q$ **then**21:      Assign $X_{n}.{Pos}_{d}$ with $N_{q}^{Neu}\in \left\{N_{min}^{Neu},N_{max}^{Neu}\right\}$ for $\;q=1,\ldots ,N_{max}^{FC}$;22:    **else if** $d==5N_{max}^{Conv}+N_{max}^{FC}+3$ **then**23:      Assign $X_{n}.{Pos}_{d}$ with $LH^{Opt}\in \left\{LH_{min}^{Opt},LH_{max}^{Opt}\right\}$;24:    **else if** $d==5N_{max}^{Conv}+N_{max}^{FC}+4$ **then**25:      Assign $X_{n}.{Pos}_{d}$ with $LH^{LR}\in \left\{LH_{min}^{LR},LH_{max}^{LR}\right\}$;26:    **else if** $d==5N_{max}^{Conv}+N_{max}^{FC}+5$ **then**27:      Assign $X_{n}.{Pos}_{d}$ with $LH^{Int}\in \left\{LH_{min}^{Int},LH_{max}^{Int}\right\}$;28:    **else if** $d==5N_{max}^{Conv}+N_{max}^{FC}+6$ **then**29:      Assign $X_{n}.{Pos}_{d}$ with $LH^{L2}\in \left\{LH_{min}^{L2},LH_{max}^{L2}\right\}$;30:    
**end if**
31:  
**end for**
32:  Fitness evaluation of $X_{n}.Pos$ as $X_{n}.Er$ using **Algorithm 2**;33:  **if** $X_{n}.Err<X^{Teacher}.Err$ **then**
34:    $X^{Teacher}.Pos\leftarrow X_{n}.Pos$, $X^{Teacher}.Err\leftarrow X_{n}.Err$; 35:  
**end if**
36:**end for****Output:** $\;P=\left[X_{1},\ldots ,X_{n},\ldots .,X_{N}\right]$, $X^{Teacher}$

Following the initialization of the position vector, denoted as $X_{n}.Pos$, for each *n*-th learner, the fitness evaluation process, as elaborated in the subsequent section, computes the learner’s fitness with respect to the classification error, indicated as $X_{n}.Err$. This initialization procedure is iteratively applied to all *N* learners, yielding an initial population denoted as $\;P=\left[X_{1},\ldots ,X_{n},\ldots .,X_{N}\right]$. The teacher is selected among these learners based on their fitness value, aiming for the lowest classification error. The position vector and fitness value of the teacher are denoted as $X^{Teacher}.Pos$ and $X^{Teacher}.Err$, respectively.

### 3.3. Fitness Evaluation of ETLBOCBL-CNN

To assess each ETLBOCBL-CNN learner’s fitness, a two-step process is employed as detailed in Algorithm 2. In the first step, a potential CNN architecture is constructed and trained using the training set. The second step evaluates this trained CNN architecture using the validation set. The fitness of each learner is quantified by measuring the classification error of its respective CNN architecture, where lower error values indicate superior fitness. In this paper, ETLBOCBL-CNN’s objective is to discover the CNN model capable of achieving the minimum classification errors when solving given datasets.

The CNN model’s configuration is determined by the network and learning hyperparameters decoded from its corresponding position vector,$\;X_{n}.Pos$. These hyperparameters encompass $N^{Conv}$, $N_{l}^{Fil}$, $S_{l}^{Ker}$, $P_{l}^{Pool}$, $S_{l}^{Pool}$, $S_{l}^{Str}$, $N^{FC}$, $N_{q}^{Neu}$, $LH^{Opt}$, $LH^{LR}$, $LH^{Int}$ and $LH^{L2}$, with $\;l=1,\ldots ,N^{Conv}$ and $q=1,\ldots ,N^{FC}$. Additionally, the CNN architecture is inserted with a fully connected layer containing an output neuron number matching the number of classes, denoted as $C^{num}$, for classification purposes.

Referring to the learning hyperparameter $LH^{Int}$, which is decoded from $X_{n}.{Pos}_{d}$ with $d=5N_{max}^{Conv}+N_{max}^{FC}+5$, a weight initializer is selected. This initializer is responsible for initializing the trainable parameters of all the convolutional and fully connected layers within the CNN. These weight parameters are denoted as $\varpi =\left\{\varpi_{1},\varpi_{2},\ldots \right\}$. Define ${ℛ}^{train}$ as the training dataset, which contains $\left|{ℛ}^{train}\right|$ samples and is used to train the potential CNN architecture constructed by every learner. To train each CNN architecture, multiple training steps of $\tau^{train}$ are executed with a predefined batch size of $S^{batch}$. During these training steps, the training data are input into the network in batches, i.e.,

(4) $$\tau^{train}=\frac{\left|{ℛ}^{train}\right|}{S^{batch}}$$

Next, an optimizer is chosen based on the learning hyperparameter $LH^{Opt}$ decoded from $X_{n}.{Pos}_{d}$ with $d=5N_{max}^{Conv}+N_{max}^{FC}+3$. This optimizer is employed to train the compiled CNN across a predetermined epoch number $\varepsilon^{train}$, performed on $\tau^{train}$ batches of data obtained from ${ℛ}^{train}$. At each *i*-th training step, where $i=1,\ldots ,\tau^{train}$, the cross-entropy loss function of CNN is obtained as $f\left(\varpi ,{ℛ}_{i}^{train}\;\right)$ based on the current weight parameters $\varpi =\left\{\varpi_{1},\varpi_{2},\ldots \right\}$ and the *i*-th batch of data, ${ℛ}_{i}^{train}$. Let $R^{L}$ represents the learning rate determined based on the learning hyperparameter $LH^{LR}$ decoded from $X_{n}.{Pos}_{d}$ with $d=5N_{max}^{Conv}+N_{max}^{FC}+4$. Additionally, $\nabla f\left(\varpi ,{ℛ}_{i}^{train}\right)$ refers to the gradient of cross-entropy loss. The new weight parameters $\;\varpi^{new}=\left\{\varpi_{1}^{new},\varpi_{2}^{new},\ldots \right\}$ for the CNN model are then updated as follows:

(5) $$\;\varpi^{new}=\varpi -R^{L}\nabla f\left(\varpi ,{ℛ}_{i}^{train}\right)$$

The performance of a CNN is assessed with a validation dataset ${ℛ}^{valid}$ of size $\left|{ℛ}^{valid}\right|$ after the training. This evaluation process is carried out in $\tau^{valid}$ steps, i.e.,

(6) $$\tau^{valid}=\frac{\left|{ℛ}^{valid}\right|}{S^{batch}}$$

In every *j*-th step of evaluation, various batches from the validation dataset, ${ℛ}_{j}^{valid}$, are utilized to assess the trained CNN models. This results in distinct classification errors, denoted as $Err\_Batch_{j}$, where $j=1,\ldots ,\tau^{valid}$. The mean classification error of the trained CNN model, considering all $\tau^{valid}$ batches of data in ${ℛ}^{valid}$, is calculated to derive the fitness value of each *n*-th learner, i.e., $X_{n}.Err$, as follows:

(7) $$X_{n}.Err=\frac{1}{\tau^{valid}}\overset{\tau^{valid}}{\underset{j=1}{\sum }}Err\_Batch_{j}$$

Finding the best CNN architecture for a given dataset with ETLBOCBL-CNN poses a considerable challenge given the time-consuming nature of exhaustive training and evaluation for each potential solution. While exploring numerous alternatives is crucial for enhancing solutions in MSA-based methods like ETLBOCBL-CNN, the exhaustive training of each learner on ${ℛ}^{train}$ with a large $\varepsilon^{train}$ is often impractical due to the substantial computational load involved. To address this challenge, a fitness approximation method is employed. It involves training the potential CNN architecture represented by each learner using a reduced training epoch (e.g., $\varepsilon^{train}=1$) during fitness evaluation. This approach, while potentially leading to less precise evaluations, significantly alleviates the computational burden. The primary aim of the selection operator is to identify the next generation of the population through fair comparisons among the learners, rather than achieving precise fitness evaluations for each learner. Additionally, a potential CNN architecture demonstrating superior performance in the initial epochs is more likely to exhibit a competitive classification error in the final stage. Upon completing the search process with ETLBOCBL-CNN, the optimal CNN architecture, constructed based on network and learning hyperparameters decoded from the teacher solution, can be thoroughly trained with a higher $\varepsilon^{train}$ to obtain its final classification error.
**Algorithm 2:** Fitness Evaluation of ETLBOCBL-CNN**Inputs:** $X_{n}.Pos$, ${ℛ}^{train}$, ${ℛ}^{valid}$, $S^{batch}$, $\varepsilon^{train}$, $R^{L}$, $C^{num}$01:Construct a candidate CNN architecture based on the network and learning hyperparameters decoded from $X_{n}.Pos$ and insert a fully connected layer with $C^{num}$ output neurons;02:Compute $\tau^{train}$ and $\tau^{valid}$ using Equations (4) and (6), respectively; 03:Generate the initial weights of the CNN model as $\;\varpi =\left\{\varpi_{1},\varpi_{2},\ldots \right\}$ using the selected weight initializer;04:**for** ε=1 to $\varepsilon^{train}$ **do**05:        **for** i=1 to $\tau^{train}$ **do**06:                   Calculate $f\left(\varpi ,{ℛ}_{i}^{train}\;\;\right)$ of CNN model;07:                   Update the weights $\;\varpi^{new}=\left\{\varpi_{1}^{new},\varpi_{2}^{new},\ldots \right\}$ based on Equation (5);08:        
**end for**
09:**end for**10:**for** j=1 to $\tau^{valid}$ **do**11:        Classify the ${ℛ}_{j}^{valid}$ dataset using the trained CNN model;12:        Record the classification errors for solving the ${ℛ}_{j}^{valid}$ dataset as $Err\_Batch_{j}$;13:**end for**14:Calculate $X_{n},Err$ of the candidate CNN architecture built from $X_{n}.Pos$ with Equation (7);**Output:**
 $X_{n}.Err$


### 3.4. Modified Teacher Phase of ETLBOCBL-CNN

The original TLBO’s teacher phase leverages both the teacher solution and the population mean to guide learners towards the global optimum, as expressed in Equation (1). Although this guidance is beneficial in the initial stages of optimization, it can lead to stagnation in later generations as diversity among exemplars diminishes. Consequently, when confronted with intricate tasks, TLBO may yield suboptimal results due to premature convergence. To address these issues, the modified teacher phase in ETLBOCBL-CNN introduces the concept of competency-based learning. This approach involves categorizing learners based on their competence levels and employing various predominant learners, alongside the teacher solution and population mean, to direct their search processes. Consequently, the modified teacher phase in ETLBOCBL-CNN can preserve swarm diversity while enhancing guidance to the population during the quest for the global optimum.

#### 3.4.1. Construction of Mean Network Architecture Represented by Population Mean

The initial step of ETLBOCBL-CNN’s modified teacher phase involves computing the population mean, denoted as $\bar{X}.Mean$. This population mean encompasses the network and learning hyperparameters of the mean network architecture based on the position vectors of all learners within the population, represented as $\;P=\left[X_{1},\ldots ,X_{n},\ldots .,X_{N}\right]$. Specifically, each dimension *d* of the population mean, which corresponds to network or learning hyperparameters, is calculated by averaging the position vectors of all learners along that dimension, as follows:

(8) $$\bar{X}.Mean_{d}=\frac{1}{N}\overset{N}{\underset{n=1}{\sum }}X_{n}.Pos_{d}$$

**Figure 6:**
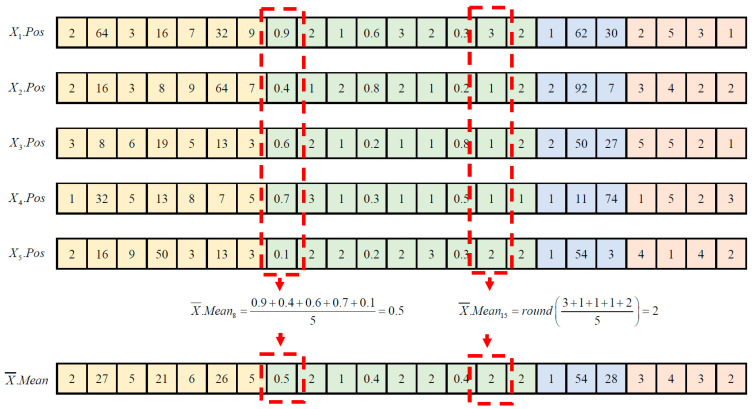
Visual representation of calculating $\bar{X}.Mean$ in ETLBOCBL-CNN.

To quantize the network and learning hyperparameters into integer values, a rounding operator, denoted as Round(·), is applied to all dimensional components of $\bar{X}.Mean$. Notably, this operation excludes hyperparameters stored in $d=2N_{max}^{Conv}+3l-1$, where $l=1,\ldots ,N_{max}^{Conv}$, as these values signify the selection probability of the pooling layer connected to the *l*-th convolutional layer. For a visual illustration of the $\bar{X}.Mean$ calculation using Equation (8), with N=5, $N_{max}^{Conv}=3$ and $N_{max}^{FC}=2$, please refer to Figure 6. Detailed pseudocode for computing the mean network architecture, as represented by $\bar{X}.Mean$, is provided in Algorithm 3.


**Algorithm 3:** Computation of Mean Network Architecture Represented by Population Mean**Input:** $\;P=\left[X_{1},\ldots ,X_{n},\ldots .,X_{N}\right]$, N, D01:$\bar{X}.Mean\leftarrow \emptyset$;02:**for** d=1 to *D* **do**03:  Compute $\bar{X}.Mean_{d}$ using Equation (8);04:  **if** $d\neq 2N_{max}^{Conv}+3l-1$ with $\;l=1,\ldots ,N_{max}^{Conv}$ **do**05:    $\bar{X}.Mean_{d}\leftarrow Round\left(\bar{X}.Mean_{d}\right)$;06:  
**end if**
07:
**end for**
**Output:**
 $\bar{X}.Mean$



#### 3.4.2. Construction of New CNN Architecture Using Competency-Based Learning

When dealing with complex optimization problems like automatic network architecture design using MSAs, it is vital to maintain diversity within the population to prevent premature convergence and becoming stuck in local optima. Simultaneously, achieving rapid convergence to the global optimum with limited computational resources is equally important. Balancing these conflicting requirements poses a significant challenge. To address this challenge, competency-based learning is introduced into the modified teacher phase of ETLBOCBL-CNN. This strategy aims to strike a balance between exploration and exploitation searches for optimal performance in solving automatic network architecture design problems. It draws inspiration from mixed-ability classrooms in modern education, where teaching is tailored to students with varying cognitive abilities. Similarly, the proposed competency-based learning recognizes that different learners have diverse potentials for exploring and exploiting the search space. Therefore, they are categorized into groups based on their competency levels, quantified through fitness values.

After calculating the population mean ($\bar{X}.Mean$), ETLBOCBL-CNN learners are sorted based on their fitness values, $X_{n}.Err$, in ascending order for n=1,…,N. All learners in the sorted $\;P=\left[X_{1},\ldots ,X_{n},\ldots .,X_{N}\right]$ are then divided into *G* groups. Each learner’s group index, *g*, is stored in $X_{n}.Grp$, where 1≤g≤G. Define $S^{Group}$ as the maximum number of learners assigned to each group (i.e., group size), and the group index *g* for each *n*-th learner is calculated as follows:

(9) $$X_{n}.Grp=g=ceil\left(\frac{n}{S^{Group}}\right)$$

where  ceil(·) is a ceiling operator that rounds up a value to the nearest integer. In Equation (9), the group index *g* of each *n*-th learner is explicitly assigned to $X_{n}.Grp$ based on *n* and $S^{Group}$. The calculation of $X_{n}.Grp$ is as follows:

(10) $$X_{n}.Grp=\{\begin{array}{cc} 1 & \mathrm{if}\;\;1\leq n\leq \;S^{Group}\\ 2 & if\;\;\;S^{Group}+1\leq n\leq \;2S^{Group}\\ 3 & if\;\;2S^{Group}+1\leq n\leq \;3S^{Group}\\ \vdots & \vdots \\ g & if\;\;\left(g-1\right)S^{Group}+1\leq n\leq \;gS^{Group}\\ \vdots & \vdots \\ G-1, & if\;\;\left(G-2\right)S^{Group}+1\leq n\leq \;\left(G-1\right)S^{Group}\\ G, & if\;\;\left(G-1\right)S^{Group}+1\leq n\leq \;GS^{Group} \end{array}$$

**Figure 7:**
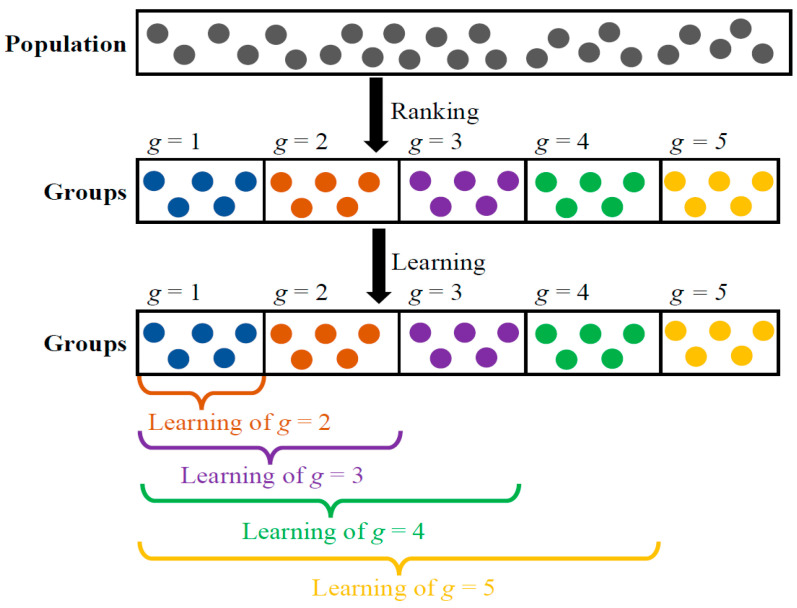
Visualization of the idea of competency-based learning introduced into the ETLOCBL-CNN’s modified teacher phase. Color dots refer to learners assigned to different groups.

Equation (10) indicates that learners with better fitness values (i.e., lower $X_{n}.Err$) are assigned smaller group indices *g* to their $X_{n}.Grp$. For instance, the top-performing $S^{Group}$ learners with population indices in the range of $n=1,\ldots ,S^{Group}$ have group index g=1 assigned to their $X_{n}.Grp$, while the bottom-performing $S^{Group}$ learners with population indices of $n=\left(G-1\right)S^{Group},\ldots ,GS^{Group}$ have group index  g=G assigned to their $X_{n}.Grp$. Learners in superior groups with smaller indices are assumed to possess more valuable information for guiding the population towards the global optimum. Conversely, learners in inferior groups with larger indices play a crucial role in exploring the search space and diverting learners away from local optima. In other words, learners in different groups exhibit varying levels of exploration and exploitation capabilities, with smaller *g* values indicating a more exploitative nature and larger *g* values indicating a stronger inclination towards exploration. Figure 7 visually represents the concept of competency-based learning within the modified teacher phase of ETLBOCBL-CNN. It highlights how learners in worse groups with larger *g* values are encouraged to learn from those predominant ones in better groups with smaller *g* values, thereby achieving a balance between exploration and exploitation searches.

Competency-based learning entails selecting predominant learners to guide the search process of learners within the solution space, in addition to the teacher solution and population mean. This selection process depends on the group index *g* assigned to each *n*-th learner in $X_{n}.Grp$, where n=1,…,N and g=1,…,G. Based on Equation (10) and Figure 7, three distinct scenarios can be identified in this learning process:

- **Scenario 1:** When $X_{n}.Grp$ is assigned to a group index of g≥3 for any *n*-th learner with $n=2S^{Group},\ldots ,GS^{Group}$, at least two groups of learners perform better than $X_{n}$.
- **Scenario 2:** When $X_{n}.Grp$ is assigned to a group index of g=2 for any *n*-th learner with $n=S^{Group},\ldots ,2S^{Group}$, only one group of learners perform better than $X_{n}$.
- **Scenario 3:** When $X_{n}.Grp$ is assigned to a group index of g=1 for any *n*-th learner with $n=1,\ldots ,S^{Group}$, no learners from any other group perform better than $X_{n}$.

In scenario 1, each *n*-th learner assigned to the *g*-th group (i.e., $X_{n}.Grp=g$) has the opportunity to learn from two predominant learners randomly selected from two superior groups ($X_{n_{r1}^{g_{r1}}}.Grp=g_{r1}$ and $X_{n_{r2}^{g_{r2}}}.Grp=g_{r2}$), where $g_{r1},\;g_{r2}\in \left\{1,g-1\right\}$ and $g_{r1}<g_{r2}<g$. The population indices for these two predominant learners can be determined as $n_{r1}^{g_{r1}}\in \left\{\left(g_{r1}-1\right)S^{Group},g_{r1}S^{Group}\right\}$ and $n_{r2}^{g_{r2}}\in \left\{\left(g_{r2}-1\right)S^{Group},g_{r2}S^{Group}\right\}$, respectively. For any *n*-th learner categorized under scenario 1, where $n=2S^{Group},\ldots ,GS^{Group}$ and g=3,….,G, a position vector $X_{n}^{off}.Pos$, representing a potential new CNN architecture, can be derived through the modified teacher phase of EETLBOCBL-CNN, as follows:

(11) $$X_{n}^{off}.Pos=X_{n}.Pos+r_{3}\left(X^{Teacher}.Pos-F^{T}\bar{X}.Mean\right)+r_{4}\left(X_{n_{r1}^{g_{r1}}}.Pos-X_{n}.Pos\right)+r_{5}\left(X_{n_{r2}^{g_{r2}}}.Pos-X_{n}.Pos\right)$$

where $r_{3},r_{4},r_{5}\in \left[0,1\right]$ are the random numbers obtained from the uniform distribution. In contrast to the original teacher phase in Equation (2), the competency-based learning in Equation (11) considers two predominant learners ($X_{n_{r1}^{g_{r1}}}$ and $X_{n_{r2}^{g_{r2}}}$) chosen from two superior groups to provide more effective guidance to the search process. These two predominant learners possess different levels of exploration and exploitation capabilities. If the predominant learner selected from group $g_{r1}$ surpasses the one from group $g_{r2}$, then predominant learner $X_{n_{r1}^{g_{r1}}}$ is more likely to lead the *n*-th learner towards the global optimum, while the other predominant learner $X_{n_{r2}^{g_{r2}}}$ is more beneficial in assisting the *n*-th learner in escaping from local optima. Furthermore, the stochastic mechanisms used to select the two superior groups (i.e., $g_{r1}$ and $g_{r2}$) and the two predominant learners (i.e., $X_{n_{r1}^{g_{r1}}}$ and $X_{n_{r2}^{g_{r2}}}$) from each selected group further contribute to the preservation of diversity within the ETLBOCBL-CNN population during the modified teacher phase.

In scenario 2, the *n*-th learner is assigned to the second group (i.e., $X_{n}.Grp=g=2$ for $n=S^{Group},\ldots ,2S^{Group}$). These learners can learn from one of two predominant learners, with population indices of $n_{r1}^{1}$ and $n_{r2}^{1}$, randomly selected from the best group with an index of g=1, where $X_{n_{r1}^{1}}.Grp=X_{n_{r2}^{1}}.Grp=1$. The population indices of these two predominant learners are determined as $n_{r1}^{1},\;n_{r2}^{1}\in \left\{1,S^{Group}\right\}$ and $n_{r1}^{1}\neq n_{r2}^{1}$. Next, the fitness values of these two predominant learners, $X_{n_{r1}^{1}}.Err$ and $X_{n_{r2}^{1}}.Err$, are compared, and only the superior one is chosen to guide the search process. For learners categorized under scenario 2, with $n=S^{Group},\ldots ,2S^{Group}$ and g=2, the position vector $X_{n}^{off}.Pos$, representing the new CNN architecture for the *n*-th learner, is determined as follows:

(12) $$X_{n}^{off}.Pos=\{\begin{array}{c} X_{n}.Pos+r_{6}\left(X^{Teacher}.Pos-F^{T}\bar{X}.Mean\right)+r_{7}\left(X_{n_{r1}^{1}}.Pos-X_{n}.Pos\right),\;\;\mathrm{if}\;X_{n_{r1}^{1}}.Err\leq X_{n_{r2}^{1}}.Err\\ X_{n}.Pos+r_{6}\left(X^{Teacher}.Pos-F^{T}\bar{X}.Mean\right)+r_{7}\left(X_{n_{r2}^{1}}.Pos-X_{n}.Pos\right),\;\;\mathrm{if}\;X_{n_{r1}^{1}}.Err>X_{n_{r2}^{1}}.Err \end{array}$$

where $r_{6},r_{7}\in \left[0,1\right]$ are the random numbers obtained from the uniform distribution.

In scenario 3, all learners assigned to the first group (i.e., $X_{n}.Grp=g=1$ for $n=1,\ldots ,S^{Group}$) are regarded as the best individuals in the population for the current generation, expected to be closer to the global optimum than other members. Given that these learners lack better exemplars from other groups to learn from, their search processes are guided solely by the teacher and the population mean, similar to the teacher phase of the original TLBO. For a learner categorized under scenario 3, where $n=1,\ldots ,S^{Group}$ and g=1, the position vector $X_{n}^{off}.Pos$, representing the new CNN architecture, is determined as follows:

(13) $$X_{n}^{off}.Pos=X_{n}.Pos+r_{8}\left(X^{Teacher}.Pos-F^{T}\bar{X}.Mean\right)$$

where $r_{8}\in \left[0,1\right]$ is a random number obtained from the uniform distribution.

The competency-based learning incorporated into the modified teacher phase of ETLBOCBL-CNN is detailed in Algorithm 4. To derive a potential new CNN architecture for each *n*-th learner (n=1,…,N and $N=GS^{Group}$), $X_{n}^{off}.Pos$ is computed using Equations (11)–(13). Subsequently, a rounding operator  Round(·) is applied to all dimensional components of $X_{n}^{off}.Pos$, except for the values stored in $d=2N_{max}^{Conv}+3l-1$, where $l=1,\ldots ,N_{max}^{Conv}$, representing the selection probability of the pooling layer connected with the *l*-th convolutional layer. The fitness of each $X_{n}^{off}.Pos$ is then assessed using Algorithm 2, producing the corresponding classification error $X_{n}^{off}.Err$. If the CNN architecture represented by $X_{n}^{off}.Pos$ yields a lower classification error than that of $X^{Teacher}.Pos$, the *n*-th offspring learner $X_{n}^{off}$ will replace the teacher solution $X^{Teacher}$. All generated offspring solutions, $X_{n}^{off}$ for n=1,…,N, are collected in the offspring population set $\;P^{off}=\left[X_{1}^{off},\ldots ,X_{n}^{off},\ldots .,X_{N}^{off}\right]$ and will be employed in the subsequent stage of the modified learner phase alongside the original population,$\;P=\left[X_{1},\ldots ,X_{n},\ldots .,X_{N}\right]$.
**Algorithm 4:** Competency-Based Learning in ETLBOCBL-CNN’s Modified Teacher Phase**Inputs:** $\;P=\left[X_{1},\ldots ,X_{n},\ldots .,X_{N}\right]$, N, D, $X^{Teacher}$, ${ℛ}^{train}$, ${ℛ}^{valid}$, $S^{batch}$, $\varepsilon^{train}$, $R^{L}$, $C^{num}$, $S^{Group}$, G01:Initialize offspring population set as $P^{off}\leftarrow \emptyset$**;**02:Calculate the population mean $\bar{X}.Mean$ using **Algorithm 3**;03: Sort all solution members of P ascendingly by referring to their fitness values $X_{n}.Err$; 04:Determine the group index *g* assigned to $X_{n}.Grp$ of all sorted learners using Equations (9) and (10); 05:**for** n=1 to N **do**06:       Initialize the *n*-th offspring learner as $X_{n}^{off}\leftarrow \emptyset$;07:       **if** $X_{n}.Grp\geq 3$ **then**08:         Randomly select two better group indices of $g_{r1},\;g_{r2}\in \left\{1,g-1\right\},$ where $g_{r1}<g_{r2}<g;$ 09:         Randomly select two predominant learners with the population indices represented as 
         $n_{r1}^{g_{r1}}\in \left\{\left(g_{r1}-1\right)S^{Group},g_{r1}S^{Group}\right\}$ and $n_{r2}^{g_{r2}}\in \left\{\left(g_{r2}-1\right)S^{Group},g_{r2}S^{Group}\right\};\;$ 10:         Calculate $X_{n}^{off}.Pos$ using Equation (11);11:       **else if** $X_{n}.Grp=2$ **then**12:         Randomly select two predominant learners from the first group (i.e., g=1) with the population 
         indices of $n_{r1}^{1},\;n_{r2}^{1}\in \left\{1,S^{Group}\right\}$ and $n_{r1}^{1}\neq n_{r2}^{1},$ where $X_{n_{r1}^{1}}.Grp=X_{n_{r2}^{1}}.Grp=1$;13:         Compare the fitness values of two predominant learners, i.e., $X_{n_{r1}^{1}}.Err$ and $X_{n_{r2}^{1}}.Err$;14:         Calculate $X_{n}^{off}.Pos$ using Equation (12);15:       **else if** $X_{n}.Grp=1$ **then**16:         Calculate $X_{n}^{off}.Pos$ using Equation (13);17:      
**end if**
18:       **for** d=1 to *D* **do**19:            **if** $d\neq 2N_{max}^{Conv}+3l-1$ with $\;l=1,\ldots ,N_{max}^{Conv}$ **then**20:             $X_{n}^{off}.Pos_{d}\leftarrow Round\left(X_{n}^{off}.Pos_{d}\;\right)$;21:          
**end if**
22:      
**end for**
23:      Perform fitness evaluation on $X_{n}^{off}.Pos$ to obtain $X_{n}^{off}.Err$ using **Algorithm 2**;24:      **if** $X_{n}^{off}.Er<X^{Teacher}.Err$ **then**
25:      $X^{Teacher}.Pos\leftarrow X_{n}^{off}.Pos$ , $X^{Teacher}.Err\leftarrow X_{n}^{off}.Err$ 26:      
**end if**
27:      $\;P^{off}\leftarrow P^{off}\cup X_{n}^{off}$; 28:**end for****Outputs:** $\;P^{off}=\left[X_{1}^{off},\ldots ,X_{n}^{off},\ldots .,X_{N}^{off}\right]$, $\;P=\left[X_{1},\ldots ,X_{n},\ldots .,X_{N}\right]$, $X^{Teacher}$

### 3.5. Modified Learner Phase of ETLBOCBL-CNN

To encourage exploration and prevent convergence toward local optima, the original TLBO employs a repelling mechanism within its single peer interaction, as seen in Equation (3). However, the effectiveness of this mechanism diminishes over iterations, particularly as the population converges. This renders it inadequate for complex problems like automatic network architecture design. Furthermore, the single peer interaction neglects the dynamics of interactions among multiple peers in a classroom, interactions that foster more efficient knowledge enhancement and the inclination of learners to preserve their original useful knowledge. To rectify these shortcomings, the modified learning phase of ETLBOCBL-CNN introduces a stochastic peer interaction scheme, aiming to enhance its performance in the discovery of optimal CNN architectures.

In the modified learner phase of ETLBOCBL-CNN, a stochastic peer learning scheme is introduced. This scheme enables each learner to interact with different peers randomly, fostering the creation of new CNN architectures. The stochastic nature of these interactions allows ETLBOCBL-CNN to escape local optima and discover more diverse solutions. Moreover, by promoting interactions among multiple peers, this phase mimics the intricate learning dynamics found in classrooms, facilitating more effective knowledge exchange and retention.

After completing the modified teacher phase, a clone population, denoted as $\;P^{clone}=\left[X_{1}^{clone},\ldots ,X_{n}^{clone},\ldots .,X_{N}^{clone}\right]$, is formed by duplicating the offspring population $\;P^{off}=\left[X_{1}^{off},\ldots ,X_{n}^{off},\ldots .,X_{N}^{off}\right]$ and sorting it in ascending order based on its fitness values, denoted as $X_{n}^{clone}.Err$ for n=1,…,N. From $P^{clone}$, two subsets of the population,$\;P^{T20}=\left[X_{1}^{T20},\ldots ,X_{n}^{T20},\ldots .,X_{0.2N}^{T20}\right]$ and $\;P^{T50}=\left[X_{1}^{T50},\ldots ,X_{n}^{T50},\ldots .,X_{0.5N}^{T50}\right]$, are created to store the top 20% and top 50% of learners from the offspring population, respectively. In the context of the stochastic peer interaction scheme, three distinct strategies are employed to update the *d*-th dimension of the position vector for each *n*-th offspring learner, $X_{n}^{off}.{Pos}_{d}$. The strategy applied is determined by a random variable rand∈[0,1]. Specifically, (a) if 0≤rand<1/3, a multiple peer interaction is triggered to update $X_{n}^{off}.{Pos}_{d}$; (b) if 1/3≤rand<2/3, a modified single peer interaction is employed to update $X_{n}^{off}.{Pos}_{d}$; and (c) if 2/3≤rand≤1, the original value of $X_{n}^{off}.{Pos}_{d}$ is retained.

Suppose two top-performing offspring learners, denoted as $X_{p}^{T20}$ and $X_{q}^{T20}$, are randomly chosen from $P^{T20}$, where p≠q≠n. If the random variable *rand* falls within the range of 0 to 1/3, the multiple peer interaction condition is triggered to update for the *d*-th component of the *n*-th learner, $X_{n}^{off}.{Pos}_{d}$, as follows:

(14) $$X_{n}^{off}.{Pos}_{d}=X_{n}^{off}.{Pos}_{d}+r_{9}\left(X_{p}^{T20}.Pos_{d}-X_{n}^{off}.Pos_{d}\right)+r_{10}\left(X_{q}^{T20}.Pos_{d}-X_{n}^{off}.Pos_{d}\right)$$

where $r_{9},r_{10}\in \left[0,1\right]$ are the random numbers obtained from the uniform distribution. 

Let $X_{r}^{T50}$ be a top-performing learner randomly chosen from $P^{T50}$, and it is utilized to update the *d*-th dimension of the *n*-th offspring learner, i.e., $X_{n}^{off}.{Pos}_{d}$, where r≠n. This update occurs through a modified single peer interaction scheme when the random variable *rand* falls in the range of 1/3 to 2/3. In this modified scheme, $X_{n}^{off}$ is attracted towards $X_{r}^{T50}$ if $X_{r}^{T50}.Err\leq X_{n}^{off}.Err$. In contrary, $X_{n}^{off}$ is repelled from $X_{r}^{T50}$ if $X_{r}^{T50}.Err>X_{n}^{off}.Err$. The formulation of this modified single peer interaction scheme for updating $X_{n}^{off}.{Pos}_{d}$ in each *d*-th dimension of the *n*-th learner is as follows:

(15) $$X_{n}^{off}.{Pos}_{d}=\{\begin{array}{c} X_{n}^{off}.Pos_{d}+r_{11}\left(X_{r}^{T50}.Pos_{d}-X_{n}^{off}.Pos_{d}\right),\;\;if\;X_{r}^{T50}.Err\leq X_{n}^{off}.Err\;\\ X_{n}^{off}.Pos_{d}+r_{11}\left(X_{n}^{off}.Pos_{d}-X_{p}^{T50}.Pos_{d}\right),\;\;if\;X_{r}^{T50}.Err>X_{n}^{off}.Err\; \end{array}$$

where $r_{11}\in \left[0,1\right]$ is a random number obtained from the uniform distribution.

The stochastic peer interaction scheme, introduced in the modified learner phase of ETLBOCBL-CNN and detailed in Algorithm 5, allows for unique updates in each dimension of the learners. These updates can involve multiple peer interactions, modified single peer interactions, or the retention of the original values, facilitating the generation of diverse candidate solutions and enhancing search capabilities. All dimensions of the updated $X_{n}^{off}.Pos$ are subject to a rounding operation using Round(·), except for those corresponding to $\;d=2N_{max}^{Conv}+3l-1$ for $\;l=1,\ldots ,N_{max}^{Conv}$ because they represent the selection probabilities of pooling layers associated with the *l*-th convolutional layer. Subsequently, Algorithm 2 is used to assess the fitness of each updated offspring learner, resulting in the computation of their classification error, $X_{n}^{off}.Err$. If the CNN architecture represented by the updated $X_{n}^{off}.Pos$ yields lower classification error than $X^{Teacher}.Pos$, the teacher solution $X^{Teacher}$ is replaced by the *n*-th updated offspring learner $X_{n}^{off}$.

### 3.6. Tri-Criterion Selection Scheme

In any optimization process employing MSAs, the choice of the selection scheme for constructing the next-generation population is pivotal. Conventional selection methods, like greedy selection and tournament selection, rely solely on the fitness values of solutions to determine their survival. For example, the original TLBO uses a greedy selection scheme to compare the fitness values of existing learners with those of new learners generated through teacher and learner phases. While these fitness-based selection schemes are straightforward to implement, they have the drawback of rejecting potentially valuable solutions with temporarily inferior fitness values that could substantially enhance the overall population quality over time. To address this limitation, the ETLBOCBL-CNN introduces a tri-criterion selection scheme. This scheme not only takes into account the fitness of learners but also factors in their diversity and improvement rate.
**Algorithm 5:** Stochastic Peer Interaction in ETLBOCBL-CNN’s Modified Teacher Phase**Inputs:** N, D, $\;P^{off}=\left[X_{1}^{off},\ldots ,X_{n}^{off},\ldots .,X_{N}^{off}\right]$, $X^{Teacher}$, ${ℛ}^{train}$, ${ℛ}^{valid}$, $S^{batch}$, $\varepsilon^{train}$, $R^{L}$, $C^{num}$01:Initialize clone population set as $P^{clone}\leftarrow \emptyset$;02:Construct $P^{clone}$ by duplicating $P^{off}$ and sorting the offspring learners ascendingly by referring to their fitness values of $X_{n}^{clone}.Err$ for n=1,…,N; 03:Construct $P^{T20}$ and $P^{T50}$ by extracting the top 20% and 50% of offspring learners stored in $P^{clone}$;04: **for** n=1 to N **do**05:   **for** d=1 to *D* **do**06:      Randomly generate rand∈[0,1] from uniform distribution; 07:      **if** 0≤rand<1/3 **then**08:        Randomly select $X_{p}^{T20}$ and $X_{q}^{T20}$ from $P^{T20}$, where p≠q≠n; 09:        Update $X_{n}^{off}.{Pos}_{d}$ using Equation (14); 10:      **else if** 1/3≤rand<2/3 **then**11:        Randomly select $X_{r}^{T50}$ from $P^{T50}$, where r≠n;12:        Update $X_{n}^{off}.{Pos}_{d}$ using Equation (15);13:       **else if** 2/3≤rand≤1 **then**14:        Retain the original value of $X_{n}^{off}.{Pos}_{d}$;15:       
**end if**
16:           **if** $d\neq 2N_{max}^{Conv}+3l-1$ with $\;l=1,\ldots ,N_{max}^{Conv}$ **then**17:       $X_{n}^{off}.{Pos}_{d}\leftarrow Round\left(X_{n}^{off}.Pos_{d}\right)$;18:       
**end if**
19:  
**end for**
20:  Perform fitness evaluation on the updated $X_{n}^{off}.Pos$ to obtain new $X_{n}^{off}.Err$ using **Algorithm 2**;21:  **if** $X_{n}^{off}.Err<X^{Teacher}.Err$ **then**
22:     $X^{Teacher}.Pos\leftarrow X_{n}^{off}.Pos$, $X^{Teacher}.Err\leftarrow X_{n}^{off}.Err$; 23:  
**end if**
24:**end for****Output:** Updated $\;P^{off}=\left[X_{1}^{off},\ldots ,X_{n}^{off},\ldots .,X_{N}^{off}\right]$ and $X^{Teacher}$ 

After completing the modified learner phase, each *n*-th offspring learner’s fitness in the updated population $\;P^{off}=\left[X_{1}^{off},\ldots ,X_{n}^{off},\ldots .,X_{N}^{off}\right]$ is compared with that of its corresponding *n*-th original learner from $\;P=\left[X_{1},\ldots ,X_{n},\ldots .,X_{N}\right]$. The improvement rate for each *n*-th offspring learner is then determined as follows:

(16) $$X_{n}^{off}.Impr=\frac{X_{n}.Err-X_{n}^{off}.Err}{∥X_{n}.Pos-X_{n}^{off}.Pos∥}$$

Here, $\left(X_{n}.Err-X_{n}^{off}.Err\right)$ in the numerator represents the change in fitness between the original and offspring learners, while $∥X_{n}.Pos-X_{n}^{off}.Pos∥$ in the denominator quantifies the Euclidean distance between these two learners. A positive $X_{n}^{off}.Impr$ indicates that the *n*-th offspring learner can yield a CNN architecture with a lower classification error compared to its original counterpart. The magnitude of $X_{n}^{off}.Impr$ plays a crucial role in assessing the effectiveness of each offspring learner in enhancing population quality. Higher values of $X_{n}^{off}.Impr$ suggest that the *n*-th offspring learner has achieved substantial improvement in classification error with a relatively small traversal in the solution space. This signifies that the offspring learner possesses valuable information for constructing a robust CNN architecture that is worth inheriting in the next generation. Notably, the improvement rate of each *n*-th original learner in P is set to $X_{n}.Impr=0$ for n=1,…,N, as these original learners serve as the baseline for comparison with their respective offspring learners.

After calculating the improvement rates for all offspring learners, the subsequent action involves creating a merged population $P^{MG}$ by combining the original $\;P=\left[X_{1},\ldots ,X_{n},\ldots ,X_{N}\right]$ with the updated $\;P^{off}=\left[X_{1}^{off},\ldots ,X_{n}^{off},\ldots .,X_{N}^{off}\right]$. The total population size of $P^{MG}$ is 2*N* and is represented as follows:

(17) $$\;P^{MG}=P\cup P^{off}=\left[X_{1}^{MG},\ldots ,X_{n}^{MG},\ldots ,X_{2N}^{MG}\right]$$

Each *n*-th solution member in $P^{MG}$, designated as $X_{n}^{MG}$, can originate from either an original learner in P or an offspring learner in $P^{off}$. These solution members in $P^{MG}$ are subsequently arranged in ascending order based on the classification error of their corresponding CNN architecture, represented by $X_{n}^{MG}.Err$. Additionally, $X_{n}^{MG}.Dis$ indicates the Euclidean distance between the CNN architecture represented by the *n*-th solution member in $P^{MG}$ (i.e., $X_{n}^{MG}.Pos$) and the current best CNN architecture, which is represented by the first solution member (i.e., $X_{1}^{MG}.Pos$), where

(18) $$X_{n}^{MG}.Dis=∥X_{n}^{MG}.Pos-X_{1}^{MG}.Pos∥\;$$

A tri-criterion selection scheme is designed to determine the next population of ETLBOCBL-CNN. This selection is based on the fitness, diversity, and improvement rate of each *n*-th solution within $P^{MG}$, denoted as $X_{n}^{MG}.Err$, $X_{n}^{MG}.Dis$, and $X_{n}^{MG}.Impr$ values, respectively, for n=1,…,2N. For the construction of the population $P^{Next}$ in the next generation, a randomly generated integer $K_{1}\in \left\{1,N\right\}$ is used. It serves to select the first $K_{1}$ solution members from $P^{MG}$, focusing on the fitness criterion. These $K_{1}$ solution members are directly selected from the subset of $P^{MG}$ with the best $X_{n}^{MG}.Err$ values.

The diversity criterion is then applied to select the next $K_{2}$ solution members for $P^{Next}$, with $K_{2}\in \left\{1,N-K_{1}\right\}$ being a randomly generated integer. The solution members in $P^{MG}$ with population indices $n=K_{1}+1,\ldots ,2N$, that were not initially chosen for $P^{Next}$, are flagged with $X_{n}^{MG}.Flag=0$, indicating their non-selection. Subsequently, the weighted fitness value $X_{n}^{MG}.WF$ is computed for the remaining $(2N-K_{1}$) solution members in $P^{MG}$ with population indices $n=K_{1}+1,\ldots ,2N$, taking into account their classification error ($X_{n}^{MG}.Err$) and diversity ($X_{n}^{MG}.Dis$) values, i.e.,

(19) $$X_{n}^{MG}.WF=\alpha \left(\frac{X_{n}^{MG}.Err-Err^{min}}{Err^{max}-Err^{min}}\right)+\left(1-\alpha \right)\left(\frac{Dis^{max}-X_{n}^{MG}.Dis}{Dis^{max}-Dis^{min}}\right)$$

The weight factor α is stochastically generated from a normal distribution with a mean of 0.9 and a standard deviation of 0.05, and it is constrained to fall within the range of 0.8 to 1.0 to maintain a balance between diversity and other selection factors. Let $Dis^{max}$ and $Dis^{min}$ represent the largest and smallest Euclidean distances measured from the best solution member $X_{1}^{MG}$, respectively, while $Err^{max}$ and $Err^{min}$ denote the worst and best fitness values observed within $P^{MG}$. Once $X_{n}^{MG}.WF$ is computed for each solution member, a binary tournament strategy is employed to randomly select two solution members, $X_{a}^{MG}$ and $X_{b}^{MG}$, from $P^{MG}$, with $a,b\in \left\{K_{1}+1,2N\right\}$, a≠b, and $X_{a}^{MG}.Flag=X_{b}^{MG}.Flag=0$. The solution member with the smaller weighted fitness value is designated as the new member of $P^{Next}$, represented as $X_{n}^{Next}$ for $n=K_{1}+1,\ldots ,K_{1}+K_{2}$, where

(20) $$X_{n}^{Next}=\{\begin{array}{c} X_{a}^{MG},\;\;\;\;\mathrm{if}\;\;X_{a}^{MG}.WF\leq X_{b}^{MG}.WF\;\\ X_{b}^{MG},\;\;\;\;\;\;\;\;\;\;\;\;\;\;\;\;\;\;\;\;\;\;\;\;\;\;\;\;\;\;\;\;\;\mathrm{otherwise} \end{array}$$

The selection process based on the diversity criterion in Equation (20) continues until all $K_{2}$ solution members are chosen for $P^{Next}$. Once a solution member of $P^{MG}$ is selected in $P^{Next}$ based on the diversity criterion, it is flagged with $X_{n}^{MG}.Flag=1$ to avoid its selection in subsequent binary tournaments, ensuring the population diversity in the next generation is not compromised.

The final $K_{3}$ solution members of $P^{Next}$ are chosen from the remaining $(2N-K_{1}$) solution members of $P^{MG}$ based on the improvement rate criterion, considering their $X_{n}^{MG}.Impr$ values for $n=K_{1}+1,\ldots ,2N$, with $K_{3}=N-K_{1}-K_{2}$. The same binary tournament strategy is applied to randomly select two solution members, $X_{e}^{MG}$ and $X_{f}^{MG}$, from $P^{MG}$, where $e,f\in \left\{K_{1}+1,2N\right\}$, e≠f, and $X_{e}^{MG}.Flag=X_{f}^{MG}.Flag=0$. The solution member with the greater improvement rate is designated as the new solution member of $P^{Next}$, i.e., $X_{n}^{Next}$, for $n=K_{1}+K_{2}+1,\ldots ,N$, where

(21) $$X_{n}^{Next}=\{\begin{array}{c} X_{e}^{MG},\;\;\;\;\mathrm{if}\;\;X_{e}^{MG}.Impr>X_{f}^{MG}.Impr\;\\ X_{f}^{MG},\;\;\;\;\;\;\;\;\;\;\;\;\;\;\;\;\;\;\;\;\;\;\;\;\;\;\;\;\;\;\;\;\;\;\;\;\;\;\;\;\mathrm{otherwise} \end{array}$$

The selection process based on the improvement rate criterion in Equation (21) continues until all $K_{3}$ solution members are included in $P^{Next}$. Similarly, any solution member of $P^{MG}$ that has been chosen for $P^{Next}$ based on the improvement rate criterion is flagged with $X_{n}^{MG}.Flag=1$ to prevent it from being selected again in the next binary tournament, maintaining population diversity.

Algorithm 6 presents the pseudocode of the proposed tri-criterion selection scheme. Unlike traditional fitness-based selection methods like greedy selection and tournament selection, the proposed selection scheme not only retains the $K_{1}$ elite solution members for $P^{Next}$ in the next iteration but also prioritizes the preservation of population diversity by simultaneously considering the diversity and improvement rate of solutions when selecting the remaining $K_{2}$ and $K_{3}$ solution members for $P^{Next}$. This approach enriches population diversity by preserving promising individuals with various solutions, thus enhancing the search process. Furthermore, it encourages the selection of solutions with a higher improvement rate, leading to quicker convergence and improved overall optimization performance. By incorporating these three criteria, the tri-criterion selection scheme empowers ETLBOCBL-CNN with a more comprehensive selection process, resulting in the selection of higher-quality solutions in the next generation.
**Algorithm 6:** Tri-Criterion Selection Scheme**Inputs:** N, $\;P=\left[X_{1},\ldots ,X_{n},\ldots ,X_{N}\right]$, $\;P^{off}=\left[X_{1}^{off},\ldots ,X_{n}^{off},\ldots .,X_{N}^{off}\right]$01:Initialize $P^{Next}\leftarrow \emptyset$;02:**for** n=1 to *N* **do**03:     Assign $X_{n}.Impr=0$ for each *n*-th original learner stored in P**;**
04:     Calculate $X_{n}^{off}.Impr$ of every *n*-th offspring learner stored in $P^{off}$ with Equation (16);05:**end for**06:Construct the merged population $P^{MG}$ using Equation (17);07:Sort the solution members in $P^{MG}$ ascendingly based on fitness values; 08:**for** n=1 to 2*N* **do**09:   Calculate $X_{n}^{MG}.Dis$ of every *n*-th solution stored in $P^{MG}$ **with** Equation (18);10:**end for**11:Randomly generate the integers of $K_{1}\in \left\{1,N\right\}$, $K_{2}\in \left\{1,N-K_{1}\right\}$ and $K_{3}=N-K_{1}-K_{2}$; 12:**for** n=1 to $K_{1}$ do /*Fitness criterion*/13:   $X_{n}^{Next}\leftarrow X_{n}^{MG}$; 14:   $\;P^{Next}\leftarrow P^{Next}\cup X_{n}^{Next}$;15:**end for**12:**for** $n=K_{1}+1$ to 2*N* **do**13:   Randomly generate α based on a normal distribution of N(0.9,0.05);14:   Restrict the value of α in between 0.8 and 1.15:   Compute the $X_{n}^{MG}.WF$ of each *n*-th solution stored in $P^{MG}$ with Equation (19);16:   Initialize the flag variable of each *n*-th solution stored in $P^{MG}$ as $X_{n}^{MG}.Flag=0$;17:**end for**18:**for** $n=K_{1}+1$ to $K_{1}+K_{2}$  do   /*Diversity criterion*/19:   Randomly select $X_{a}^{MG}$ and $X_{b}^{MG}$ from $P^{MG}$, where $a,b\in \left\{K_{1}+1,2N\right\}$, a≠b, and $X_{a}^{MG}.Flag=X_{b}^{MG}.Flag=0$.20:   Determine $X_{n}^{Next}$ with Equation (20);21:     $\;P^{Next}\leftarrow P^{Next}\cup X_{n}^{Next}$;22:   **if** $X_{a}^{MG}$ is selected as $X_{n}^{Next}$ then  /*Prevent the selection of same solution members*/23:       $X_{a}^{MG}.Flag=1$;24:   **else if** $X_{b}^{MG}$ is selected as $X_{n}^{Next}$ **then**25:       $X_{b}^{MG}.Flag=1$;26:   
**end if**
27:**end for**28:**for** $n=K_{1}+K_{2}+1$ to N  do   /*Improvement rate criterion*/29:   Randomly select $X_{e}^{MG}$ and $X_{f}^{MG}$ from $P^{MG}$, where $e,f\in \left\{K_{1}+1,2N\right\}$, e ≠f, and $X_{e}^{MG}.Flag=X_{f}^{MG}.Flag=0$.30:   Determine $X_{n}^{Next}$ using Equation (21);31:     $\;P^{Next}\leftarrow P^{Next}\cup X_{n}^{Next}$;32:   **if** $X_{e}^{MG}$ is selected as $X_{n}^{Next}$ then /*Prevent the selection of same solution members*/33:       $X_{e}^{MG}.Flag=1$;34:   **else if** $X_{f}^{MG}$ is selected as $X_{n}^{Next}$ **then**35:       $X_{f}^{MG}.Flag=1$;36:   
**end if**
37:**end for****Output:**
 $\;P^{Next}=\left[X_{1}^{Next},\ldots ,X_{n}^{Next},\ldots .,X_{N}^{Next}\right]$


### 3.7. Complete Mechanisms of ETLBOCBL-CNN

Algorithm 7 offers a comprehensive overview of the complete mechanisms within ETLBOCBL-CNN for optimizing CNN architecture concerning a specific dataset. In this context, the current iteration is stored in a counter variable t, and the predefined maximum iteration number $T^{max}$ serves as the termination criterion for ETLBOCBL-CNN. The process commences with loading the training (${ℛ}^{train}$) and validation (${ℛ}^{valid}$) datasets from the directory, followed by the initialization of the population using Algorithm 1. ETLBOCBL-CNN then proceeds to iteratively generate a new offspring population set, $P^{off}$, comprising various CNN architectures, through the modified teacher and learner phases, which are executed using Algorithms 4 and 5, respectively. Following this, the next-generation population, $P^{Next}$, is formed by applying the proposed tri-criterion selection scheme detailed in Algorithm 6 to the merged population $P^{MG}=P\cup P^{off}$. The optimization process is considered complete when $t>T^{max}$.

As mentioned earlier, the fitness evaluation process in Algorithm 2 is designed to employ a reduced epoch number $\varepsilon^{train}$ when training the CNN architecture created by each ETLBOCBL-CNN learner. While this approach can lower computational overhead, it may be insufficient for effectively addressing complex problems. Therefore, following the termination of ETLBOCBL-CNN, the CNN architecture produced by the teacher solution, designated as $X^{Teacher}.Pos$, undergoes an extensive training process. This process employs the same mechanisms as those in Algorithm 2 but utilizes a larger epoch number, $\varepsilon^{FT}$. The objective is to ensure that the CNN stemming from the teacher solution is thoroughly trained and possesses the capability to effectively tackle complex problems. Upon the completion of this full training process, comprehensive network information is returned, encompassing architecture, classification error, and the number of trainable parameters, thereby offering valuable insights into the optimized CNN architecture.
**Algorithm 7:** Proposed ETLBOCBL-CNN **Inputs:** N, D, ${ℛ}^{train}$, ${ℛ}^{valid}$, $S^{batch}$, $\varepsilon^{train}$, $\varepsilon^{FT}$, $R^{L}$, $C^{num}$, $N_{min}^{Conv}$, $N_{max}^{Conv}$, $N_{min}^{Fil}$, $N_{max}^{Fil}$, $S_{min}^{Ker}$, $S_{max}^{Ker}$, $S_{min}^{Pool}$, $S_{max}^{Pool}$, $S_{min}^{Str}$, $S_{max}^{Str}$, $N_{min}^{FC}$, $N_{max}^{FC}$, $N_{min}^{Neu}$, $N_{max}^{Neu}$, $S^{Group}$, G01:Load ${ℛ}^{train}$ and ${ℛ}^{valid}$ from the directory; 02:Initialize the population $\;P=\left[X_{1},\ldots ,X_{n},\ldots ,X_{N}\right]$ using **Algorithm 1**;03:Initialize the iteration counter as t←0; 04:**while** $t<T^{max}$ **do**05:     Generate $P^{off}$ and update $X^{Teacher}$ using modified teacher phase (**Algorithm 4**);06:     Update $P^{off}$ and $X^{Teacher}$ using modified learner phase (**Algorithm 5**);07:     Determine $P^{Next}\;$ using tri-criterion selection scheme (**Algorithm 6**);08:      
 $P\leftarrow P^{Next}\;;$
09:     t←t+1;10:**end while**11:Fully train the CNN architecture constructed from $X^{Teacher}.Pos$ with larger $\varepsilon^{FT}$ (**Algorithm 2**);**Output:** $X^{Teacher}$ and its corresponding optimal CNN architecture

## 4. Performance Evaluation of ETLBOCBL-CNN

### 4.1. Benchmark Dataset Selection

**Figure 8:**
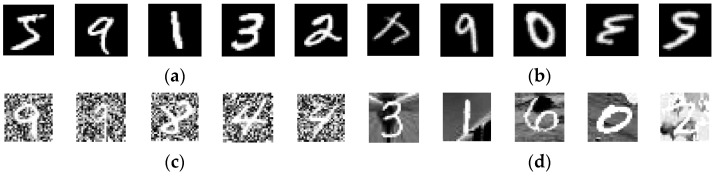

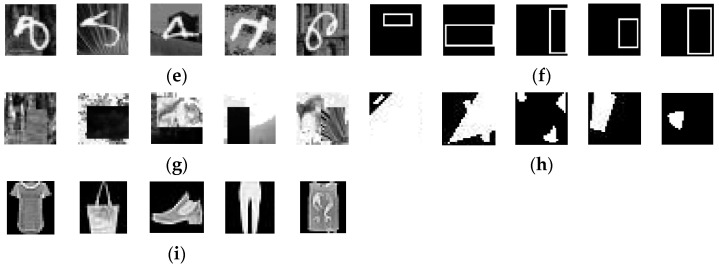
Sample images of the datasets: (**a**) MNIST, (**b**) MNIST-RD, (**c**) MNIST-RB, (**d**) MNIST-BI, (**e**) MNIST-RD + BI, (**f**) Rectangles, (**g**) Rectangles-I, (**h**) Convex, and (**i**) Fashion.

This section focuses on evaluating the image classification performance of network architectures developed using ETLBOCBL-CNN. These architectures are assessed across nine benchmark datasets: Modified National Institute of Standards and Technology (MNIST), MNIST with Rotated Digits (MNIST-RD), MNIST with Random Background (MNIST-RB), MNIST with Background Images (MNIST-BI), MNIST with Rotated Digits and Background Image (MNIST-RD + BI), Rectangles, Rectangles with Images (Rectangles-I), Convex, and Fashion. These datasets were obtained from http://www.iro.umontreal.ca/~lisa/icml2007data/ (accessed on 3 June 2023). Sample images for each dataset are visually represented in Figure 8, and Table 3 offers an overview of the selected datasets.

The selection of these nine benchmark datasets for this study is based on several justifications. Firstly, the current study prioritizes the development of an efficient MSA-based automated network architecture search method that can scale to larger datasets in the future, given the limited computing resources. As a result, this study has chosen to test the proposed ETLBOCBL-CNN exclusively on these nine datasets, which have small input sizes (28×28×1). Additionally, each of these benchmark datasets exhibits distinct characteristics related to the types of objects to be classified. This diversity makes them well-suited for evaluating the overall effectiveness of the proposed ETLBOCBL-CNN in searching for optimal CNN architectures capable of robustly classifying various types of datasets. Furthermore, these benchmark datasets are selected because different algorithms have previously reported promising results on them. This choice allows for convenient comparisons between the performance of ETLBOCBL-CNN and the algorithms that have achieved the best results on these datasets.

The MNIST dataset [70] consists of grayscale images featuring handwritten digits from 0 to 9, serving as a standard benchmark for classifier evaluation. To increase the complexity of classification tasks, four variants of the MNIST dataset are designed [71]: MNIST-RD, MNIST-RB, MNIST-BI, and MNIST-RD + BI. These variants introduce additional elements such as rotation, random background noise, background images, and combinations of rotation and background images. These enhancements are designed to test the generalization capabilities of classifiers. Notably, all four MNIST variants present imbalanced distributions between their training and testing datasets, further challenging classifiers in extracting meaningful features.

The Rectangle dataset comprises grayscale images displaying outlines of rectangles of different sizes. It is used to assess classifier performance in recognizing larger rectangles, whether in terms of height or width. On the other hand, the Rectangle-I dataset presents a more intricate challenge. It contains grayscale images of rectangles, each containing additional images within its boundaries. Solving this dataset involves the identification of image patches within the rectangle or within the background.

The Convex dataset comprises grayscale images portraying a variety of geometric shapes, encompassing both convex and non-convex forms. It serves as a means to evaluate a classifier’s capacity to recognize and differentiate between these geometric types. Finally, the Fashion dataset [72] consists of grayscale images featuring various fashion products, categorized into ten classes of items like trousers, dresses, coats, tops, bags, sneakers, sandals, ankle boots, pullovers, and shirts. Due to its elevated complexity, the Fashion dataset presents a demanding challenge for assessing classifier performance.

### 4.2. Simulation Settings

The performance of ETLBOCBL-CNN is assessed by comparing it with thirteen established machine learning and deep learning models known for high classification accuracy across eight selected datasets: MNIST, MNIST-RD, MNIST-RB, MNIST-RI, MNIST-RD + BI, Rectangle, Rectangle-I, and Convex. These algorithms include ScatNet-2 [73], LDANet-2 [74], PCANet-2 [74], RandNet-2 [74], NNet [71], CAE-1 [75], CAE-2 [75], DBN-3 [71], SAA-3 [71], SVM + Poly [71], SVM + RBF [71], EvoCNN [76], and particle-swarm-based CNN (psoCNN) [62]. Furthermore, the performance of ETLBOCBL-CNN is evaluated against thirteen additional algorithms, namely 2C1P2F, 2C1P2F + Dropout, 3C2F, 3C1P2F + Dropout, MLP 256-128-64, MLP 256-128-100, AlexNet [77], SqueezeNet [78], HOG + SVM, GRU + SVM, GRU + SVM + Dropout, psoCNN [62], and EvoCNN [76], specifically for the Fashion dataset. These algorithm results are sourced from reputable literature and publicly available code repositories.

Notably, EvoCNN and psoCNN, like ETLBOCBL-CNN, are MSA-based algorithms used for iteratively searching optimal CNN architectures within a maximum iteration limit. Most of the selected peer algorithms were manually designed for specific tasks, making them suitable for comparison with ETLBOCBL-CNN’s autonomous capability to discover optimal CNN architectures that achieve higher accuracy with fewer network parameters, with minimal human intervention. ETLBOCBL-CNN’s parameter settings conform to established conventions in the deep learning and MSA communities, as detailed in Table 4. To ensure the statistical robustness of the findings, 30 independent simulation runs of ETLBOCBL-CNN were conducted on a computer equipped with Python 3.8.5 and Nvidia GeForce RTX 3090.

### 4.3. Performance Analyses

#### 4.3.1. Comparison in Classifying the First Eight Benchmark Datasets

Table 5 presents a comprehensive overview of classification accuracies achieved by the proposed ETLBOCBL-CNN and its peer algorithms across eight challenging benchmark image datasets: MNIST, MNIST-RD, MNIST-RB, MNIST-BI, MNIST-RD + BI, Rectangles, Rectangles-I, and Convex. The accuracies are determined using the respective test datasets, ensuring a fair evaluation of generalization capabilities. For clarity, Table 5 highlights the best and second-best results for each dataset in bold and underlined, respectively. Symbols such as “(+)”, “(−)”, and “(=)” indicate whether ETLBOCBL-CNN outperforms, lags behind, or equals the peer algorithm’s classification accuracy for a specific dataset. “NA” denotes cases where dataset results are unavailable for direct comparison, as they were sourced from existing literature. To provide a concise summary of the results, the “*w*/*t*/*l*” metric conveys whether ETLBOCBL-CNN’s discovered optimal CNN architectures outperform, match, or underperform compared to peers in the *w*, *t*, and *l* datasets. Additionally, “*#BCA*” informs the number of instances where each compared algorithm achieves the highest classification accuracy across all eight benchmark image datasets.

**Figure 9:**
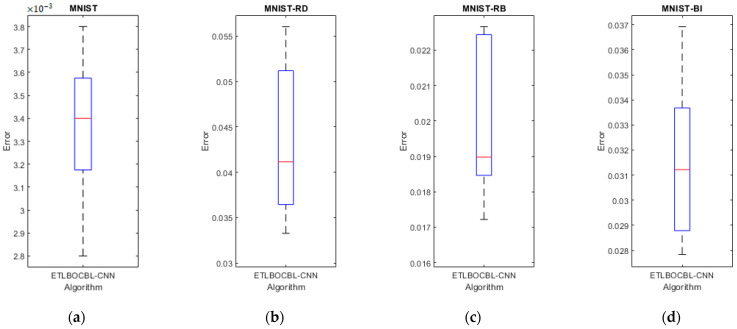

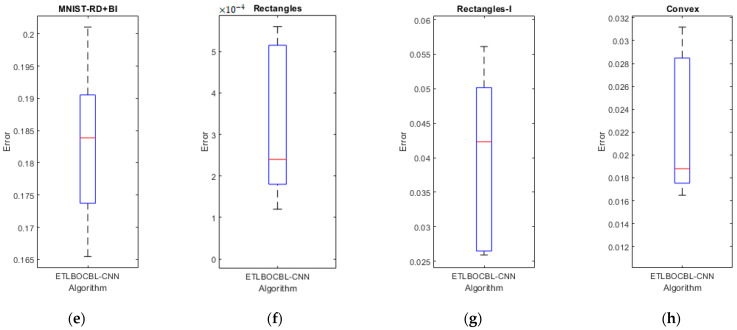
Test errors obtained by ETLBOCBL-CNN while solving the eight datasets: (**a**) MNIST, (**b**) MNIST-RD, (**c**) MNIST-RB, (**d**) MNIST-BI, (**e**) MNIST-RD + BI, (**f**) Rectangles, (**g**) Rectangles-I, and (**h**) Convex.

The remarkable performance of ETLBOCBL-CNN is evident from the results in Table 5. It attains the highest classification accuracies across various image datasets, including MNIST (99.72%), MNIST-RD (96.67%), MNIST-RB (98.28%), MNIST-BI (97.22%), MNIST-RD + BI (83.45%), Rectangles (99.99%), Rectangles-I (97.41%), and Convex (98.35%). In comparison to nine other algorithms (LDANet-2, PCANet-2, RandNet-2, NNet, DBN-3, SAA-3, SVM-Poly, SVM-RBF, and psoCNN), ETLBOCBL-CNN consistently achieves higher classification accuracies across all selected image datasets. Furthermore, ETLBOCBL-CNN demonstrates outstanding mean classification accuracies of 99.66%, 95.65%, 98.00%, 96.85%, 81.72%, 99.97%, 97.41%, and 98.35%, surpassing the performance of thirteen other algorithms in solving five out of the eight datasets: MNIST, MNIST-RD, MNIST-RB, MNIST-RD + BI, and Convex. In addition to classification accuracies, Figure 9 presents the distribution of test errors generated by ETLBOCBL-CNN for these eight datasets through boxplots, offering insights into its overall performance. These simulation results reveal that MSA-based methods like ETLBOCBL-CNN, psoCNN, and EvoCNN consistently rank among the top two best-performing algorithms for solving these image datasets. This indicates that integrating MSAs into the automated construction of optimal CNN models can be a promising alternative to manual trial-and-error network design, reducing the need for extensive human intervention. Furthermore, the excellent performance of ETLBOCBL-CNN in classifying the majority of benchmark datasets, compared to EvoCNN and psoCNN, highlights the efficacy of incorporating competency-based learning and stochastic peer interaction schemes into the modified teacher and learner phases of ETLBOCBL-CNN. These modifications strike a balance between explorative and exploitative search strategies, enabling ETLBOCBL-CNN to construct promising CNN models automatically, reducing the reliance on human expertise. The integration of a tri-criterion selection scheme in ETLBOCBL-CNN is also highly beneficial. This scheme considers criteria beyond fitness, safeguarding valuable network information within the learners. It promotes diversity and facilitates a commendable rate of fitness improvement, even when learners’ current fitness levels are temporarily inferior. Both diversity and the fitness improvement rate significantly contribute to the potential of ETLBOCBL-CNN in achieving long-term advancements in performance, especially in tackling complex problems like optimizing CNN architecture design.

To further investigate the performance disparity between ETLBOCBL-CNN and its peer algorithms when addressing the chosen image datasets, a series of non-parametric statistical analyses [79] were conducted. These analyses aimed to provide deeper insights into the classification accuracies presented in Table 5, with CAE-1 and CAE-2 excluded from the analysis due to their lack of classification accuracies for the Convex dataset. A performance comparison using the Wilcoxon signed-rank test [79] was conducted to examine the performance difference between ETLBOCBL-CNN and its peer algorithms across the eight selected datasets. The results of the Wilcoxon signed-rank test, including the $R^{+}$, $R^{-}$, *p*, and *h* values, are presented in Table 6. $R^{+}$ and $R^{-}$ represent the sum of ranks indicating the superiority and inferiority of ETLBOCBL-CNN compared to each algorithm, respectively. The *p* value serves as a measure of the minimum significance level required to detect a performance difference between two algorithms. An algorithm is considered significantly better than its peer when the *p* value falls below the threshold value of α=0.05. Additionally, the *h* value is denoted as “+”, “=”, or “−” to signify whether ETLBOCBL-CNN is significantly better, insignificantly different, or significantly worse than its peer in solving the selected datasets.

The Friedman test [79] was also conducted to comprehensively analyze the overall performance disparities between ETLBOCBL-CNN and the other peer algorithms in solving the eight image datasets. Table 7 presents the rankings of all compared algorithms based on their classification accuracies, from the best to the worst: ETLBOCBL-CNN, psoCNN, EvoCNN, LDANet-2, PCANet-2, ScatNet-2, RandNet-2, DBN-3, SAA-3, SVM + RBF, SVM + Poly, and NNet. Table 7 also highlights significant global differences among all compared algorithms, with a *p* value smaller than α=0.05. To explore the specific differences among the algorithms, three post hoc statistical analysis procedures [79], namely Bonferroni–Dunn, Holm, and Hochberg, were conducted. These procedures aimed to provide a comprehensive understanding of the discrepancies between the compared algorithms and ETLBOCBL-CNN, which served as the control algorithm. Table 8 presents the *z* values, unadjusted *p* values, and adjusted *p* values (APVs) obtained from the post hoc procedures. It is worth noting that APVs smaller than  α=0.05, denoted in boldface, confirm the significant improvement of ETLBOCBL-CNN over NNet, SVM + Poly, SVM + RBF, SAA-3, and DBN-3. Moreover, the Holm and Hochberg procedures verify the significant improvement of ETLBOCBL-CNN over RandNet-2 and ScatNet-2.

#### 4.3.2. Comparison in Classifying the MNIST-Fashion Datasets

Table 9 displays the classification accuracies and the total number of trainable parameters for network architectures generated by ETLBOCBL-CNN and 13 other peer algorithms. Notably, MSA-based methods, such as ETLBOCBL-CNN, consistently exhibit remarkable performance when applied to the MNIST-Fashion dataset. The results in Table 5 highlight the ability of MSAs to automatically construct optimal CNN models with high classification accuracy while maintaining lower network complexity, applicable to various image classification tasks. In the context of the MNIST-Fashion dataset, the proposed ETLBOCBL-CNN achieves the second-best classification accuracy of 93.70%. EvoCNN and psoCNN, two other MSA-based algorithms, secure the highest and third-best accuracies of 94.53% and 92.81%, respectively. These outcomes demonstrate the effectiveness of MSA-based approaches, including ETLBOCBL-CNN, in achieving state-of-the-art performance for image classification tasks.

ETLBOCBL-CNN achieves a classification accuracy in solving the MNIST-Fashion dataset that is slightly lower than that of EvoCNN, with a negligible difference of less than 1%. Notably, the CNN architecture produced by ETLBOCBL-CNN demonstrates remarkable efficiency, featuring only 0.843 million trainable parameters. This parameter number is significantly lower, being approximately 87.38% and 67.33% less than the parameter numbers of EvoCNN (6.68 million) and psoCNN (2.58 million), respectively. These findings reveal that ETLBOCBL-CNN strikes a favorable balance between classification accuracy and network complexity when designing optimal CNN architectures for specific classification tasks. In recent years, the integration of automated smart systems into various aspects of daily life, including identity recognition systems, traffic monitoring systems, and mobile navigation systems, has become increasingly prevalent. However, many of these technologies are embedded in mobile or edge devices with limited computational power and power resources. Consequently, there is a growing demand for deep learning models that are resource-efficient to support the development of these emerging technologies. In this context, the proposed ETLBOCBL-CNN, with its capacity to create networks with reduced complexity, emerges as a highly desirable solution for the advancement of diverse mobile smart systems.

When comparing ETLBOCBL-CNN to other established algorithms such as 2C1P2F + Dropout, 3C1P2F + Dropout, AlexNet, and MLP 256-128-100 in solving the MNIST-Fashion dataset, the performance of the latter algorithms is notably inferior. These algorithms are characterized by networks with a considerably larger number of trainable parameters, ranging from 3.27 million to an extensive 60 million. This observation implies that many manually crafted deep learning models often feature an excess of trainable parameters that do not significantly enhance classification accuracy. Instead, they result in unnecessary consumption of computational resources. In contrast, ETLBOCBL-CNN distinguishes itself by achieving competitive performance not only on the MNIST-Fashion dataset but also across the other eight datasets. What sets ETLBOCBL-CNN apart is its capacity to deliver exceptional classification results without the need for data augmentation techniques or overly complex network structures. This is achieved by initializing the learners with simpler network architectures, which leads to faster convergence rates during the search process. Furthermore, ETLBOCBL-CNN illustrates the feasibility of achieving state-of-the-art classification performance using a simpler network structure, highlighting the effectiveness and efficiency of the proposed approach.

#### 4.3.3. Optimal Network and Learning Hyperparameters Obtained by ETLBOCBL-CNN

Table 10 presents the optimal network architectures and learning hyperparameters identified by ETLBOCBL-CNN to achieve the highest classification accuracy across the selected image datasets. It is noteworthy that the optimal CNN architectures consistently feature a single fully connected layer. This aligns with recent studies [80], suggesting that CNN models with a single fully connected layer tend to outperform those with multiple fully connected layers. Interestingly, it is also observed that the inclusion of a pooling layer between successive convolution layers is not mandatory for achieving the best classification accuracy, as seen in results from the MNIST, MNIST-RD, MNIST-RB, MNIST-BI, MNIST-RD + BI, Rectangles, and Convex datasets.

Furthermore, Table 10 highlights that the effectiveness of the network architectures generated by ETLBOCBL-CNN for different image datasets relies heavily on the thoughtful selection of learning hyperparameters during the training process. The optimal combinations of learning hyperparameters are determined by considering the specific characteristics of the given image datasets and the configuration of the discovered network architectures. The results in Table 10 verify ETLBOCBL-CNN’s capacity to minimize network complexity by eliminating redundant layers and parameters while maintaining high performance. This unique capability of ETLBOCBL-CNN offers compelling evidence of its ability to autonomously design optimal network architectures for various classification tasks, even with minimal prior knowledge of the problem domains.

### 4.4. Discussion

#### 4.4.1. Impact of Proposed Modifications in ETLBOCBL-CNN

As demonstrated in the prior subsection, ETLBOCBL-CNN has displayed commendable performance in generating optimal CNN architectures that effectively address nine selected datasets, delivering superior classification accuracy. The effectiveness of ETLBOCBL-CNN stems from three main modifications: (a) the competency-based learning within the modified teacher phase, (b) the stochastic peer interaction scheme within the modified learner phase, and (c) the tri-criterion selection scheme. This subsection delves into a detailed discussion of the individual contributions of each modification, conducted through an ablation study to gauge their specific impacts on ELTBOCLB-CNN when addressing the optimization problem of automatic network architecture design.

To establish a baseline, the ablation study incorporates a method called TLBO-CNN. To gauge the effects of the three major modifications introduced in this study, three variants of ETLBOCBL-CNN are investigated: ETLBOCBL-CNN-v1, ETLBOCBL-CNN-v2, and ETLBOCBL-CNN-v3. In ETLBOCBL-CNN-v1, competency-based learning is employed within its modified teacher phase, retaining the original learner phase. In contrast, ETLBOCBL-CNN-v2 maintains the original teacher phase but integrates the stochastic peer interaction scheme into its modified learner phase. ETLBOCBL-CNN-v3 incorporates both competency-based learning and stochastic peer interaction schemes in the modified teacher and learner phases, respectively. It is notable that these ETLBOCBL-CNN variants use the greedy-based selection scheme, isolating the impact of the proposed tri-criterion selection scheme when compared to the complete ETLBOCBL-CNN.

The performance of TLBO-CNN and all ETLBOCBL-CNN variants in the automated generation of CNN architectures for the nine selected datasets is presented in Table 11. The reported metrics include classification accuracy and the number of trainable parameters, indicative of network complexity. ETLBOCBL-CNN variants consistently outperform the baseline TLBO-CNN. They exhibit the capability to generate CNN architectures with reduced network complexity while achieving higher classification accuracy for most datasets. This demonstrates the efficacy of the modified teacher and learner phases in addressing automatic network architecture design challenges. Compared to TLBO-CNN’s original teacher phase, ETLBO-CNN’s competency-based learning mechanism in the modified teacher phase excels in preserving population diversity. It accomplishes this by guiding each learner using personalized and high-performing peers selected from different superior groups. This approach enhances robustness against premature convergence, maintaining rapid convergence towards promising solution regions based on personalized guidance. Additionally, the stochastic peer interaction scheme in ETLBOCBL-CNN’s modified learner phase offers more flexibility compared to TLBO-CNN’s original learner phase. It accurately emulates the complex learning dynamics observed in a classroom. ETLBOCBL-CNN learners employ diverse learning strategies and more effective knowledge-sharing mechanisms during this phase, leading to the discovery of diverse CNN architectures. The multiple peer interaction and modified single peer interaction schemes facilitate different levels of exploratory searches, fostering knowledge sharing and collaborative learning among peers. Simultaneously, the stochastic peer interaction scheme promotes exploitation by retaining valuable network information acquired during previous learning processes. The proper balancing between exploration and exploitation searches attained in both the modified teacher and learner phases of ETLBOCBL-CNN has improved the effectiveness of CNN architecture search.

Table 11 shows varying performance between ELTBOCBL-CNN-v1 and ETLBOCBL-CNN-v2 in solving automatic network design problems. ETLBOCBL-CNN-v2 generally outperforms ETLBOCBL-CNN-v1 in classification accuracy and network complexity across four datasets: MNIST, MNIST-RD, MNIST-RD + BI, and Convex. Conversely, ETLBOCBL-CNN-v1 excels in MNIST-Fashion with higher accuracy and lower network complexity compared to ETLBOCBL-CNN-v2. For the other four datasets (MNIST-RB, MNIST-BI, Rectangles, and Rectangle-I), ETLBOCBL-CNN-v1 performs better in network complexity, while ETLBOCBL-CNN-v2 exhibits a competitive edge in classification accuracy. These observations verify the unique contributions of competency-based learning and stochastic peer interaction to ETLBOCBL-CNN in solving automatic network design problems. Additionally, ETLBOCBL-CNN-v3 produces more competitive classification accuracy and network complexity across all nine datasets compared to ETLBOCBL-CNN-v1 and ETLBOCBL-CNN-v2, affirming the synergistic effects of the competency-based learning in the modified teacher phase and the stochastic peer interaction in the modified learner phase. These factors assist ETLBOCBL-CNN in searching for optimal CNN architectures with high accuracy and low complexity when solving the given tasks.

Finally, Table 11 highlights that the complete ETLBOCBL-CNN outperforms ETLBOCBL-CNN-v3 by generating CNN architectures with higher accuracy and lower complexity across all nine datasets. While there are marginal differences in classification accuracy across most datasets, complete ETLBOCBL-CNN excels notably in reducing network complexity compared to ETLBOCBL-CNN-v3. Unlike TLBO-CNN and the three ETLBOCBL-CNN variants, which rely solely on the fitness criterion, complete ETLBOCBL-CNN employs a tri-criterion selection scheme. This scheme considers fitness, diversity, and fitness improvement when deciding the survival of population members in the next generation. The proposed tri-criterion selection scheme allows learners with relatively lower fitness but greater diversity or potential for improvement to survive, enhancing population diversity and resilience against premature convergence. In the context of automatic network architecture design, the performance comparisons in Table 11 demonstrate the effectiveness of the tri-criterion selection scheme in discovering high-performing, low-complexity CNN architectures for effective classification tasks.

#### 4.4.2. Qualitative Complexity Analysis of ETLBOCBL-CNN

In this subsection, a detailed Big O analysis is conducted to examine the time complexity of both the proposed ETLBOCBL-CNN and the baseline TLBO-CNN. It is important to note that both methods share the same time complexity for fitness evaluation when addressing automatic network architecture design tasks with identical benchmark datasets. Let *N* represent the population size and *D* the problem dimensionality. TLBO-CNN exhibits a time complexity O(ND) for generating the initial population and creating new solutions through both the teacher and learner phases. Consequently, the overall time complexity for TLBO-CNN in each iteration is O(ND) in the worst-case scenario.

ETLBOCBL-CNN shares a similar time complexity to TLBO-CNN during population initialization, also at O(ND). However, the overall time complexity of ETLBOCBL-CNN is governed by the three key modifications introduced, namely (a) competency-based learning in the modified teacher phase, (b) the stochastic peer interaction scheme in the modified learner phase, and (c) the tri-criterion selection scheme.

In each iteration of the modified teacher phase, the computation of population means, $\bar{X}.Mean$, using Equation (8) incurs a time complexity of O(ND). The sorting process that arranges all learners based on their fitness values in ascending order has a time complexity of O(NlogN). Additionally, the competency-based learning mechanism, employed to calculate the values of $X_{n}^{off}$ for all *N* learners using Equations (11), (12), and (13), results in a time complexity of O(ND). Since O(ND) exhibits a higher growth rate than O(NlogN), the overall time complexity of the modified teacher phase is O(ND) per iteration.

A time complexity of O(NlogN) is required for sorting the offspring learners by their fitness levels in each iteration of the modified learner phase. The creation of $P^{T20}$ and $P^{T50}$, containing the top 20% and 50% of offspring learners, demands a time complexity of O(N) per iteration. During the computation of $X_{n}^{off}$ using the stochastic peer interaction scheme, including the multiple peer interaction as defined in Equation (14), modified single peer interaction as defined in Equation (15), and the knowledge retention mechanism, a time complexity of O(ND) is incurred for all *N* learners. Hence, the total time complexity of the modified teacher phase in ETLBOCBL-CNN remains O(ND) per iteration.

For the tri-criterion selection scheme, the calculation of $X_{n}^{off}.Impr$ for all *N* offspring learners in $P^{off}$ using Equation (16) incurs a time complexity of O(ND) per iteration, while generating the merged population $P^{MG}$ using Equation (17) requires O(N) per iteration. Subsequently, the sorting of population members in $P^{MG}$ has a time complexity of O(NlogN) per iteration. Additionally, the procedures for calculating $X_{n}^{MG}.Dis$ using Equation (18) and $X_{n}^{MG}.WF$ using Equation (19) for all learners in each iteration result in time complexities of O(ND) and O(N), respectively. When constructing $P^{Next}$ for the subsequent iteration of ETLBOCBL-CNN based on fitness, diversity, and improvement rate criteria, a time complexity of O(N) per iteration is incurred. The collective time complexity of the tri-criterion selection scheme in ETLBOCBL-CNN amounts to O(ND) per iteration.

The time complexity analyses conducted for each key modification of ETLBOCBL-CNN reveal that the overall time complexity for each iteration is O(ND) in the worst-case scenario. This implies that the time complexity of ETLBOCBL-CNN is inherently tied to the population size (*N*) and the problem’s dimensional size (*D*). In this study, the total dimensional size is calculated as $D=5N_{max}^{Conv}+N_{max}^{FC}+6$. Consequently, the time complexity of ETLBOCBL-CNN is contingent on the upper limit values of the convolutional layer number ($N_{max}^{Conv}$) and the fully connected layer number ($N_{max}^{FC}$). These values can be thoughtfully determined based on the input image size used for classification tasks.

#### 4.4.3. Quantitative Complexity Analysis of ETLBOCBL-CNN

In this subsection, a quantitative complexity analysis of ETLBOCBL-CNN is performed. Specifically, Table 12 presents the computational time required by ETLBOCBL-CNN to search for an optimal CNN architecture for each of the selected benchmark datasets with the best classification accuracy. The computation time for automatically searching for the optimal CNN architecture across the nine benchmark datasets varies from 1013.88 s (approximately 17 min) to 14,227.21 s (approximately 4 h). The simulation results in Table 12 highlight a significant advantage of ETLBOCBL-CNN: its ability to generate high-performing yet computationally efficient CNN architectures, making it a valuable solution for resource-constrained environments, such as mobile or edge devices, where efficient resource utilization is paramount. 

ETLBOCBL-CNN has a wide range of potential applications, including medical image analysis, natural language processing, autonomous vehicles, recommendation systems, environmental monitoring, and more. The simulation results confirm ETLBOCBL-CNN as a robust and effective approach for automated CNN architecture design. It excels in achieving a balance between classification accuracy and network complexity, positioning it as a promising solution for various image classification tasks.

The computational times reported in Table 12 are based on utilizing the proposed ETLBOCBL-CNN to address the nine benchmark datasets, each with an input size of 28×28×1. As outlined in the previous subsection’s Big O analysis, the overall time complexity of ETLBOCBL-CNN inherently depends on $N_{max}^{Conv}$ and $N_{max}^{FC}$. These two upper limit values need to be thoughtfully set based on the input image size used for specific classification tasks. While the primary focus of current study is on designing ETLBOCBL-CNN for the automatic search of optimal CNN architectures tailored to specific datasets, it is crucial to discuss the scalability of this method, particularly when applied to real-world applications involving large-scale problems and diverse datasets.

Scaling up deep learning methods to handle more extensive datasets and complex problems invariably presents substantial challenges, and ETLBOCOL-CNN is no exception. A primary challenge pertains to computational resources. Larger datasets and more complex architectures demand increased training times, memory resources, and processing power. This could potentially restrict the use of ETLBOCOL-CNN in scenarios with limited computing infrastructure. Furthermore, as data volume increases in larger-scale problems, challenges related to model generalization and overfitting may arise. With growing data complexity, maintaining model robustness becomes increasingly critical.

However, amid these scalability challenges, ETLBOCBL-CNN offers several advantages. The proposed modifications such as competency-based learning, the stochastic peer interaction scheme, and the tri-criterion selection scheme have demonstrated their potentials to enhance generalization and robustness when dealing with diverse datasets. The automated architecture design process of ETLBOCBL-CNN remains a valuable asset for accelerating model development, reducing human intervention, and improving performance. To tackle these scalability issues, mitigation strategies can be employed. Techniques like parallel processing, distributed computing, or specialized hardware utilization can significantly reduce training times and efficiently handle more extensive datasets. It is imperative to explore these options to fully unlock the potential of ETLBOCBL-CNN.

## 5. Conclusions

In this study, ETLBOCBL-CNN is presented as an innovative method for optimizing CNN architectures, offering an automated approach to efficiently design optimal network structures for classification tasks across various complexities. This method incorporates an efficient solution encoding scheme to discover valid and novel CNN architectures. It optimizes both network hyperparameters and learning. To enhance ETLBOCBL-CNN’s performance, this paper introduces a competency-based learning concept that categorizes learners according to their fitness values, guiding each learner’s search process not only with the teacher solution and population mean but also with guidance from other predominant learners. This promotes diverse exploration and prevents convergence toward local optima. Moreover, a stochastic peer interaction scheme is integrated into ETLBOCBL-CNN’s learner phase, enhancing its robustness against local optima through collaborative learning among learners. This scheme enables effective knowledge sharing and retention, engaging single or multiple peer learners. To overcome the limitations of greedy selection, ETLBOCBL-CNN introduces a tri-criterion selection scheme, which assesses learners’ survival in the next generation by considering their fitness, diversity, and improvement rate. This scheme promotes diversity and ensures continued fitness improvement, even when current fitness levels are temporarily inferior. It safeguards valuable network information within learners and enhances population quality over the long term.

The proposed ETLBOCBL-CNN’s performance is assessed across nine image datasets and compared with state-of-the-art methods. The results demonstrate that ETLBOCBL-CNN consistently achieves competitive classification accuracies, often surpassing or matching the performance of existing peer algorithms. This solidifies ETLBOCBL-CNN as a robust and effective approach for automated CNN architecture design, effectively balancing classification accuracy and network complexity. Its adaptability to various datasets enhances its versatility. Future research can explore incorporating advanced network blocks like DenseNet and ResNet into ETLBOCBL-CNN to generate advanced CNN architectures. Additionally, formulating the automatic network architecture design as a multi-objective optimization problem opens possibilities for a multi-objective ETLBOCBL-CNN capable of satisfying diverse stakeholder requirements, including factors like inference speed and network parameters.

## Figures and Tables

**Table 1:** Upper and lower boundary limits of network and learning hyperparameters.

Section	Hyperparameter	Value
Convolution	Lower limit of convolutional layers, $N_{min}^{Conv}$	1
	Upper limit of convolutional layers, $N_{max}^{Conv}$	3
	Lower limit of filter numbers, $N_{min}^{Fil}$	3
	Upper limit of filter numbers, $N_{max}^{Fil}$	256
	Lower limit of kernel size, $S_{min}^{Ker}$	3 × 3
	Upper limit of kernel size, $S_{max}^{Ker}$	9 × 9
Pooling	Lower limit of pooling size, $S_{min}^{Pool}$	1 × 1
	Upper limit of pooling size, $S_{max}^{Pool}$	3 × 3
	Lower limit of stride size, $S_{min}^{Str}$	1 × 1
	Upper limit of stride size, $S_{max}^{Str}$	2 × 2
Fully connected	Lower limit of fully connected layer number, $N_{min}^{FC}$	1
	Upper limit of fully connected layer number, $N_{max}^{FC}$	2
	Lower limit of neuron numbers, $N_{min}^{Neu}$	1
	Upper limit of neuron numbers, $N_{max}^{Neu}$	300
Training	Lower limit of integer indices to select optimizer type, $LH_{min}^{Opt}$	1
	Upper limit of integer indices to select optimizer type, $LH_{max}^{Opt}$	5
	Lower limit of integer indices to select learning rate, $LH_{min}^{LR}$	1
	Upper limit of integer indices to select learning rate, $LH_{max}^{LR}$	5
	Lower limit of integer indices to select initializer type, $LH_{min}^{Int}$	1
	Upper limit of integer indices to select initializer type, $LH_{max}^{Int}$	5
	Lower limit of integer indices to select L2-regularizer, $LH_{min}^{L2}$	1
	Upper limit of integer indices to select L2-regularizer, $LH_{max}^{L2}$	5

**Table 2:** Integer indices assigned for the selection of training hyperparameters.

Integer Index	Types of Training Hyperparameters			
	$\mathrm{Optimizer}\;\mathrm{Type}\;LH^{Opt}$	Learning Rate $LH^{LR}$	Initializer Type $LH^{Int}$	L2-Regularizer $LH^{L2}$
1	Adadelta [63]	0.0001	Glorot Normal [64]	0.001
2	Adagrad [65]	0.0005	Glorot Uniform [64]	0.005
3	Adam [66]	0.001	He Normal [67]	0.01
4	Adamax [68]	0.005	He Uniform [67]	0.05
5	SGD [69]	0.01	Random Uniform	0.1

**Table 3:** An overview of nine image datasets utilized for performance evaluation.

Dataset	Total No. of Dataset	No. of Training Dataset	No. of Testing Dataset	Input Size	No. of Output Classes
MNIST	70,000	60,000	10,000	28×28×1	10
MNIST-RD	62,000	12,000	50,000	28×28×1	10
MNIST-RB	62,000	12,000	50,000	28×28×1	10
MNIST-BI	62,000	12,000	50,000	28×28×1	10
MNIST-RD + BI	62,000	12,000	50,000	28×28×1	10
Rectangles	51,200	1200	50,000	28×28×1	2
Rectangles-I	62,000	12,000	50,000	28×28×1	2
Convex	58,000	8000	50,000	28×28×1	2
Fashion	70,000	60,000	10,000	28×28×1	10

**Table 4:** Parameter settings of ETLBOCBL-CNN used for performance evaluation.

Parameter	Value
Maximum iteration number, $T^{max}$	10
Population size, *N*	20
Dimension size, *D*	23
Lower limit of convolutional layer numbers, $N_{min}^{Conv}$	1
Upper limit of convolutional layer numbers, $N_{max}^{Conv}$	3
Lower limit of filter number, $N_{min}^{Fil}$	3
Upper limit of filter number, $N_{max}^{Fil}$	256
Lower limit of kernel size, $S_{min}^{Ker}$	3×3
Upper limit of kernel size, $S_{max}^{Ker}$	9×9
Lower limit of pooling size, $S_{min}^{Pool}$	1×1
Upper limit of pooling size, $S_{max}^{Pool}$	3×3
Lower limit of stride size, $S_{min}^{Str}$	1×1
Upper limit of stride size, $S_{max}^{Str}$	2×2
Lower limit of of fully connected layer numbers, $N_{min}^{FC}$	1
Upper limit of fully connected layer numbers, $N_{max}^{FC}$	2
Lower limit of neuron numbers, $N_{min}^{Neu}$	1
Upper limit of neuron numbers, $N_{max}^{Neu}$	300
Lower limit of integer index to select optimizer type, $LH_{min}^{Opt}$	1
Upper limit of integer index to select optimizer type, $LH_{max}^{Opt}$	5
Lower limit of integer index to select learning rate, $LH_{min}^{LR}$	1
Upper limit of integer index to select learning rate, $LH_{max}^{LR}$	5
Lower limit of integer index to select initializer type, $LH_{min}^{Int}$	1
Upper limit of integer index to select initializer type, $LH_{max}^{Int}$	5
Lower limit of integer index to select L2-regularizer, $LH_{min}^{L2}$	1
Upper limit of integer index to select L2-regularizer, $LH_{max}^{L2}$	5
Inclusion of batch normalization	Yes
Dropout rate	0.5
Epoch number for the fitness evaluation of learner, $\varepsilon^{train}$	1
Epoch number for the full training of the best learner returned, $\varepsilon^{FT}$	100

**Table 5:** Classification accuracies obtained by ETLBOCBL-CNN and its peers when tackling the eight selected datasets.

Algorithm	MNIST	MNIST-RD	MNIST-RB	MNIST-BI	MNIST-RD + BI
ScatNet-2	98.73% (+)	92.52% (+)	87.70% (+)	81.60% (+)	49.52% (+)
LDANet-2	98.95% (+)	92.48% (+)	93.19% (+)	87.58% (+)	61.46% (+)
PCANet-2	98.60% (+)	91.48% (+)	93.15% (+)	88.45% (+)	64.14% (+)
RandNet-2	98.75% (+)	91.53% (+)	86.53% (+)	88.35% (+)	56.31% (+)
NNet	95.31% (+)	81.89% (+)	79.96% (+)	72.59% (+)	37.84% (+)
CAE-1	98.60% (+)	95.48% (+)	93.19% (+)	87.58% (+)	61.46% (+)
CAE-2	97.52% (+)	90.34% (+)	89.10% (+)	84.50% (+)	54.77% (+)
DBN-3	96.89% (+)	89.70% (+)	93.27% (+)	83.69% (+)	52.61% (+)
SAA-3	96.54% (+)	89.70% (+)	88.72% (+)	77.00% (+)	48.07% (+)
SVM + Poly	96.31% (+)	84.58% (+)	83.38% (+)	75.99% (+)	43.59% (+)
SVM + RBF	96.97% (+)	88.89% (+)	85.42% (+)	77.49% (+)	44.82% (+)
EvoCNN	98.82% (+)	94.78% (+)	97.20% (+)	95.47% (+)	64.97% (+)
psoCNN	99.51% (+)	94.56% (+)	97.61% (+)	96.87% (+)	81.05% (+)
ETLBOCBL-CNN (Best)	**99.72%**	**96.67%**	**98.28%**	**97.22%**	**83.45%**
ETLBOCBL-CNN (Mean)	99.66%	95.65%	98.00%	96.85%	81.72%
Algorithm	Rectangles	Rectangles-I	Convex	*w*/*t*/*l*	*#BCA*
ScatNet-2	**99.99% (=)**	91.98% (+)	93.50% (+)	7/1/0	1
LDANet-2	99.86% (+)	83.80% (+)	92.78% (+)	8/0/0	0
PCANet-2	99.51% (+)	86.61% (+)	95.81% (+)	8/0/0	0
RandNet-2	99.91% (+)	83.00% (+)	94.55% (+)	8/0/0	0
NNet	92.84% (+)	66.80% (+)	67.75% (+)	8/0/0	0
CAE-1	99.86% (+)	83.80% (+)	NA	7/0/0	0
CAE-2	98.46% (+)	78.00% (+)	NA	7/0/0	0
DBN-3	97.39% (+)	77.50% (+)	81.37% (+)	8/0/0	0
SAA-3	97.59% (+)	75.95% (+)	81.59% (+)	8/0/0	0
SVM + Poly	97.85% (+)	75.95% (+)	80.18% (+)	8/0/0	0
SVM + RBF	97.85% (+)	75.96% (+)	80.87% (+)	8/0/0	0
EvoCNN	**99.99% (=)**	94.97% (+)	95.18% (+)	7/1/0	1
psoCNN	99.93% (+)	96.03% (+)	97.74% (+)	8/0/0	0
ETLBOCBL-CNN (Best)	**99.99%**	**97.41%**	**98.35%**	NA	8
ETLBOCBL-CNN (Mean)	99.97%	96.02%	97.76%	NA	NA

**Table 6:** Wilcoxon signed-rank test results comparing ETLBOCBL-CNN with its peers.

ETLBOCBL-CNN vs.	$R^{+}$	$R^{-}$	*p* Value	*h* Value
ScatNet-2	28.0	0.0	$1.42\times 10^{-2}$	+
LDANet-2	36.0	0.0	$9.58\times 10^{-3}$	+
PCANet-2	36.0	0.0	$9.58\times 10^{-3}$	+
RandNet-2	36.0	0.0	$9.58\times 10^{-3}$	+
NNet	36.0	0.0	$8.37\times 10^{-3}$	+
DBN-3	36.0	0.0	$9.58\times 10^{-3}$	+
SAA-3	36.0	0.0	$9.58\times 10^{-3}$	+
SVM + Poly	36.0	0.0	$9.58\times 10^{-3}$	+
SVM + RBF	36.0	0.0	$9.58\times 10^{-3}$	+
EvoCNN	28.0	0.0	1$.42\times 10^{-3}$	+
psoCNN	36.0	0.0	$8.37\times 10^{-3}$	+

**Table 7:** Average ranking and corresponding *p* values obtained from the Friedman test.

Algorithm	Ranking	Chi-Square Statistic	*p* Value
ScatNet-2	5.8125	76.658654	$0.00\times 10^{0}$
LDANet-2	5.2500		
PCANet-2	5.2500		
RandNet-2	6.0000		
NNet	12.0000		
DBN-3	7.9375		
SAA-3	9.0625		
SVM + Poly	10.5625		
SVM + RBF	9.4375		
EvoCNN	3.0625		
psoCNN	2.5000		
ETLBOCBL-CNN	1.1250		

**Table 8:** Adjusted *p* values (APVs) calculated using the three post hoc procedures.

ETLBOCBL-CNN vs.	*z*	Unadjusted *p*	Bonferroni–Dunn *p*	Holm *p*	Hochberg *p*
Nnet	$6.03\times 10^{0}$	$0.00\times 10^{0}$	$0.00\times 10^{0}$	$0.00\times 10^{0}$	$0.00\times 10^{0}$
SVM + Poly	$5.23\times 10^{0}$	$0.00\times 10^{0}$	$2.00\times 10^{-6}$	$2.00\times 10^{-6}$	$2.00\times 10^{-6}$
SVM + RBF	$4.61\times 10^{0}$	$4.00\times 10^{-6}$	$4.40\times 10^{-5}$	$3.60\times 10^{-5}$	$3.60\times 10^{-5}$
SAA-3	$4.40\times 10^{0}$	$1.10\times 10^{-5}$	$1.17\times 10^{-4}$	$8.50\times 10^{-5}$	$8.50\times 10^{-5}$
DBN-3	$3.78\times 10^{0}$	$1.58\times 10^{-4}$	$1.73\times 10^{-3}$	$1.10\times 10^{-3}$	$1.10\times 10^{-3}$
RandNet-2	$2.70\times 10^{0}$	$6.85\times 10^{-3}$	$7.53\times 10^{-2}$	$4.11\times 10^{-2}$	$4.11\times 10^{-2}$
ScatNet-2	$2.60\times 10^{0}$	$9.32\times 10^{-3}$	$1.02\times 10^{-1}$	$4.66\times 10^{-2}$	$4.66\times 10^{-2}$
LDANet-2	$2.29\times 10^{0}$	$2.21\times 10^{-2}$	$2.43\times 10^{-1}$	$8.85\times 10^{-2}$	$6.64\times 10^{-2}$
PCANet-2	$2.29\times 10^{0}$	$2.21\times 10^{-2}$	$2.43\times 10^{-1}$	$8.85\times 10^{-2}$	$6.64\times 10^{-2}$
EvoCNN	$1.07\times 10^{0}$	$2.82\times 10^{-1}$	$3.11\times 10^{0}$	$5.65\times 10^{-1}$	$4.66\times 10^{-1}$
psoCNN	$7.63\times 10^{-1}$	$4.66\times 10^{-1}$	$4.90\times 10^{0}$	$5.65\times 10^{-1}$	$4.66\times 10^{-1}$

**Table 9:** Performance evaluation of the proposed ETLBOCBL-CNN alongside its peer algorithms when tackling the MNIST-Fashion dataset.

Algorithm	Classification Accuracy	No. of Trainable Parameters
Human Performance ^1^	83.50%	NA
2C1P2F + Dropout ^1^	91.60%	$3.27\times 10^{6}$
2C1P ^1^	92.50%	$1.00\times 10^{5}$
3C2F ^1^	90.70%	NA
3C1P2F + Dropout ^1^	92.60%	$7.14\times 10^{6}$
GRU + SVM ^1^	88.80%	NA
GRU + SVM + Dropout	89.70%	NA
HOG + SVM ^1^	92.60%	NA
AlexNet [77]	89.90%	$6.00\times 10^{7}$
SqueezeNet-200 [78]	90.00%	$5.00\times 10^{5}$
MLP 256-128-64 ^1^	90.00%	$4.10\times 10^{4}$
MLP 256-128-100 ^1^	88.33%	$3.00\times 10^{6}$
EvoCNN [76]	94.53%	$6.68\times 10^{6}$
psoCNN [62]	92.81%	$2.58\times 10^{6}$
ETLBOCBL-CNN (Best)	93.70%	$8.43\times 10^{5}$
ETLBOCBL-CNN (Mean)	93.12%	$1.95\times 10^{6}$

^1^ https://github.com/zalandoresearch/fashion-mnist (accessed on 3 June 2023).

**Table 10:** Optimal network and learning hyperparameters derived by ETLBOCBL-CNN to solve each selected image dataset with the highest classification accuracy.

Dataset	Layers	Network Hyperparameters	Learning Hyperparameters
MNIST	Convolutional	$N_{1}^{Fil}=231$, $S_{1}^{Ker}=9\times 9$	$LH^{Opt}=3$ (‘Adam’)
	Maximum Pooling	$S_{1}^{Pool}=2\times 2$, $S_{1}^{Str}=1\times 1$	$LH^{LR}=3$ (‘0.001’)
	Convolutional	$N_{2}^{Fil}=101$, $S_{2}^{Ker}=9\times 9$	$LH^{Int}=1$ (‘Glorot Normal’)
	Convolutional	$N_{3}^{Fil}=97$, $S_{3}^{Ker}=9\times 9$	$LH^{L2}=1$ (‘0.001’)
	Fully Connected	$N_{1}^{Neu}=10$	
MNIST-RD	Convolutional	$N_{1}^{Fil}=96$, $S_{1}^{Ker}=9\times 9$	$LH^{Opt}=3$ (‘Adam’)
	Convolutional	$N_{2}^{Fil}=47$, $S_{2}^{Ker}=9\times 9$	$LH^{LR}=3$ (‘0.001’)
	Convolutional	$N_{3}^{Fil}=125$, $S_{3}^{Ker}=9\times 9$	$LH^{Int}=1$ (‘Glorot Normal’)
	Average Pooling	$S_{3}^{Pool}=3\times 3$, $S_{3}^{Str}=1\times 1$	$LH^{L2}=2$ (‘0.005’)
	Fully Connected	$N_{1}^{Neu}=10$	
MNIST-RB	Convolutional	$N_{1}^{Fil}=47$, $S_{1}^{Ker}=3\times 3$	$LH^{Opt}=3$ (‘Adam’)
	Convolutional	$N_{2}^{Fil}=112$, $S_{2}^{Ker}=9\times 9$	$LH^{LR}=4$ (‘0.005’)
	Convolutional	$N_{3}^{Fil}=65$, $S_{3}^{Ker}=9\times 9$	$LH^{Int}=2$ (‘Glorot Uniform’)
	Average Pooling	$S_{3}^{Pool}=3\times 3$, $S_{3}^{Str}=1\times 1$	$LH^{L2}=3$ (‘0.01’)
	Fully Connected	$N_{1}^{Neu}=10$	
MNIST-BI	Convolutional	$N_{1}^{Fil}=76$, $S_{1}^{Ker}=3\times 3$	$LH^{Opt}=3$ (‘Adam’)
	Convolutional	$N_{2}^{Fil}=137\;$, $S_{2}^{Ker}=6\times 6$	$LH^{LR}=4$ (‘0.005’)
	Convolutional	$N_{3}^{Fil}=181$, $S_{3}^{Ker}=7\times 7$	$LH^{Int}=2$ (‘Glorot Uniform’)
	Maximum Pooling	$S_{3}^{Pool}=3\times 3$, $S_{3}^{Str}=2\times 2$	$LH^{L2}=3$ (‘0.01’)
	Fully Connected	$N_{1}^{Neu}=10$	
MNIST-RD + BI	Convolutional	$N_{1}^{Fil}=48$, $S_{1}^{Ker}=5\times 5$	$LH^{Opt}=3$ (‘Adam’)
	Convolutional	$N_{2}^{Fil}=63\;$, $S_{2}^{Ker}=7\times 7$	$LH^{LR}=3$ (‘0.001’)
	Maximum Pooling	$S_{2}^{Pool}=3\times 3$, $S_{2}^{Str}=1\times 1$	$LH^{Int}=2$ (‘Glorot Uniform’)
	Convolutional	$N_{3}^{Fil}=108\;$, $S_{3}^{Ker}=8\times 8$	$LH^{L2}=3$ (‘0.01’)
	Average Pooling	$S_{3}^{Pool}=2\times 2$, $S_{3}^{Str}=1\times 1$	
	Fully Connected	$N_{1}^{Neu}=10$	
Rectangles	Convolutional	$N_{1}^{Fil}=234$, $S_{1}^{Ker}=9\times 9$	$LH^{Opt}=3$ (‘Adam’)
	Convolutional	$N_{2}^{Fil}=89$, $S_{2}^{Ker}=9\times 9$	$LH^{LR}=2$ (‘0.0005’)
	Maximum Pooling	$S_{2}^{Pool}=3\times 3$, $S_{2}^{Str}=1\times 1$	$LH^{Int}=2$ (‘Glorot Uniform’)
	Convolutional	$N_{3}^{Fil}=85$, $S_{3}^{Ker}=9\times 9$	$LH^{L2}=1$ (‘0.001’)
	Average Pooling	$S_{3}^{Pool}=3\times 3$, $S_{3}^{Str}=2\times 2$	
	Fully Connected	$N_{1}^{Neu}=2$	
Rectangles-I	Convolutional	$N_{1}^{Fil}=74$, $S_{1}^{Ker}=3\times 3$	$LH^{Opt}=4$ (‘Adamax’)
	Maximum Pooling	$S_{1}^{Pool}=2\times 2$, $S_{1}^{Str}=1\times 1$	$LH^{LR}=3$ (‘0.001’)
	Convolutional	$N_{2}^{Fil}=161$, $S_{2}^{Ker}=9\times 9$	$LH^{Int}=1$ (‘Glorot Normal’)
	Average Pooling	$S_{2}^{Pool}=1\times 1$, $S_{2}^{Str}=2\times 2$	$LH^{L2}=4$ (‘0.05’)
	Convolutional	$N_{3}^{Fil}=207$, $S_{3}^{Ker}=9\times 9$	
	Maximum Pooling	$S_{3}^{Pool}=3\times 3$, $S_{3}^{Str}=1\times 1$	
	Fully Connected	$N_{1}^{Neu}=2$	
Convex	Convolutional	$N_{1}^{Fil}=136$, $\;S_{1}^{Ker}=9\times 9$	$LH^{Opt}=4$ (‘Adamax’)
	Maximum Pooling	$S_{1}^{Pool}=3\times 3$, $S_{1}^{Str}=1\times 1$	$LH^{LR}=3$ (‘0.001’)
	Convolutional	$N_{2}^{Fil}=118$,$\;S_{2}^{Ker}=9\times 9$	$LH^{Int}=1$ (‘Glorot Normal’)
	Convolutional	$N_{3}^{Fil}=197$,$\;S_{3}^{Ker}=9\times 9$	$LH^{L2}=3$ (‘0.01’)
	Fully Connected	$N_{1}^{Neu}=2$	
MNIST-Fashion	Convolutional	$N_{1}^{Fil}=164$, $\;S_{1}^{Ker}=3\times 3$	$LH^{Opt}=3$ (‘Adam’)
	Maximum Pooling	$S_{1}^{Pool}=2\times 2$, $S_{1}^{Str}=1\times 1$	$LH^{LR}=3$ (‘0.001’)
	Convolutional	$N_{2}^{Fil}=96$, $\;S_{2}^{Ker}=3\times 3$	$LH^{Int}=3$ (‘He Normal’)
	Fully Connected	$N_{1}^{Neu}=10$	$LH^{L2}=1$ (‘0.001’)

**Table 11:** Ablation study to gauge the impacts of proposed modifications in ETLBOCBL-CNN.

Dataset	Metric	TLBO-CNN	ETLBOCBL-CNN Variants			
			v1	v2	v3	Complete
MNIST	Accuracy	98.54%	98.66%	98.74%	99.01%	**99.72%**
	Complexity	12.10 M	10.68 M	8.94 M	4.71 M	**3.41 M**
MNIST-RD	Accuracy	94.66%	96.34%	96.46%	96.58%	**96.67%**
	Complexity	10.10 M	3.92 M	2.34 M	2.19 M	**1.67 M**
MNIST-RB	Accuracy	96.91%	97.90%	97.92%	98.04%	**98.28%**
	Complexity	7.23 M	3.85 M	6.16 M	5.67 M	**1.47 M**
MNIST-BI	Accuracy	95.53%	96.34%	96.37%	97.10%	**97.22%**
	Complexity	5.02 M	2.07 M	2.45 M	1.98 M	**1.90 M**
MNIST-RD + BI	Accuracy	77.58%	78.19%	82.20%	82.74%	**83.45%**
	Complexity	3.71 M	6.11 M	4.95 M	2.22 M	**1.26 M**
Rectangles	Accuracy	99.68%	99.71%	99.79%	99.90%	**99.99%**
	Complexity	12.60 M	6.24 M	11.51 M	2.76 M	**2.34 M**
Rectangles-I	Accuracy	95.71%	97.24%	97.36%	97.37%	**97.41%**
	Complexity	6.63 M	**2.02 M**	6.05 M	3.47 M	5.51 M
Convex	Accuracy	95.20%	97.12%	97.55%	97.71%	**98.35%**
	Complexity	1.54 M	3.55 M	2.59 M	1.51 M	**1.46 M**
MNIST-Fashion	Accuracy	91.89%	92.91%	91.99%	93.12%	**93.70%**
	Complexity	4.31 M	3.12 M	3.44 M	2.97 M	**0.84 M**

**Table 12:** Computational time incurred to find the optimal CNN architectures to solve each dataset.

Datasets	Computational Time (s)
MNIST	5945.89
MNIST-RD	2851.13
MNIST-RB	3087.26
MNIST-BI	3317.30
MNIST-RD + BI	2735.13
Rectangles	1013.88
Rectangles-I	4132.82
Convex	1380.63
MNIST-Fashion	14,227.21

## Data Availability

The data will be provided upon reasonable request.

## References

[1] Ji S., Xu W., Yang M., Yu K. (2012). 3D convolutional neural networks for human action recognition. IEEE Trans. Pattern Anal. Mach. Intell..

[2] Wang P., Li Z., Hou Y., Li W. Action recognition based on joint trajectory maps using convolutional neural networks. Proceedings of the 24th ACM International Conference on Multimedia.

[3] Jayanthi J., Jayasankar T., Krishnaraj N., Prakash N., Sagai Francis Britto A., Vinoth Kumar K. (2021). An intelligent particle swarm optimization with convolutional neural network for diabetic retinopathy classification model. J. Med. Imaging Health Inform..

[4] Goel T., Murugan R., Mirjalili S., Chakrabartty D.K. (2021). OptCoNet: An optimized convolutional neural network for an automatic diagnosis of COVID-19. Appl. Intell..

[5] Müller A., Karathanasopoulos N., Roth C.C., Mohr D. (2021). Machine learning classifiers for surface crack detection in fracture experiments. Int. J. Mech. Sci..

[6] Sharma N., Jain V., Mishra A. (2018). An analysis of convolutional neural networks for image classification. Procedia Comput. Sci..

[7] Tang Y., Huang Z., Chen Z., Chen M., Zhou H., Zhang H., Sun J. (2023). Novel visual crack width measurement based on backbone double-scale features for improved detection automation. Eng. Struct..

[8] Wu Z., Tang Y., Hong B., Liang B., Liu Y. (2023). Enhanced precision in dam crack width measurement: Leveraging advanced lightweight network identification for pixel-level accuracy. Int. J. Intell. Syst..

[9] Wu F., Yang Z., Mo X., Wu Z., Tang W., Duan J., Zou X. (2023). Detection and counting of banana bunches by integrating deep learning and classic image-processing algorithms. Comput. Electron. Agric..

[10] Yu N., Xu Q., Wang H. (2019). Wafer defect pattern recognition and analysis based on convolutional neural network. IEEE Trans. Semicond. Manuf..

[11] Liu Y., Sun Y., Xue B., Zhang M., Yen G.G., Tan K.C. (2021). A survey on evolutionary neural architecture search. IEEE Trans. Neural Netw. Learn. Syst..

[12] Wistuba M., Rawat A., Pedapati T. (2019). A survey on neural architecture search. arXiv.

[13] Liu C., Zoph B., Neumann M., Shlens J., Hua W., Li L.-J., Fei-Fei L., Yuille A., Huang J., Murphy K. Progressive neural architecture search. Proceedings of the European Conference on Computer Vision (ECCV).

[14] Pham H., Guan M., Zoph B., Le Q., Dean J. Efficient neural architecture search via parameters sharing. Proceedings of the International Conference on Machine Learning.

[15] Jaafra Y., Laurent J.L., Deruyver A., Naceur M.S. (2019). Reinforcement learning for neural architecture search: A review. Image Vis. Comput..

[16] Zhao J., Zhang R., Zhou Z., Chen S., Jin J., Liu Q. (2021). A neural architecture search method based on gradient descent for remaining useful life estimation. Neurocomputing.

[17] Kandasamy K., Neiswanger W., Schneider J., Poczos B., Xing E.P. (2018). Neural architecture search with bayesian optimisation and optimal transport. Adv. Neural Inf. Process. Syst..

[18] Zhou H., Yang M., Wang J., Pan W. Bayesnas: A bayesian approach for neural architecture search. Proceedings of the International Conference on Machine Learning.

[19] Camero A., Wang H., Alba E., Bäck T. (2021). Bayesian neural architecture search using a training-free performance metric. Appl. Soft Comput..

[20] Ahmad M., Abdullah M., Moon H., Yoo S.J., Han D. (2020). Image classification based on automatic neural architecture search using binary crow search algorithm. IEEE Access.

[21] Oyelade O.N., Ezugwu A.E. (2021). A bioinspired neural architecture search based convolutional neural network for breast cancer detection using histopathology images. Sci. Rep..

[22] Arman S.E., Deowan S.A. (2022). IGWO-SS: Improved grey wolf optimization based on synaptic saliency for fast neural architecture search in computer vision. IEEE Access.

[23] Zoph B., Le Q.V. (2016). Neural architecture search with reinforcement learning. arXiv.

[24] Liu H., Simonyan K., Yang Y. (2018). Darts: Differentiable architecture search. arXiv.

[25] Yu H., Peng H., Huang Y., Fu J., Du H., Wang L., Ling H. (2022). Cyclic differentiable architecture search. IEEE Trans. Pattern Anal. Mach. Intell..

[26] Xue Y., Qin J. (2022). Partial connection based on channel attention for differentiable neural architecture search. IEEE Trans. Ind. Inform..

[27] Cai Z., Chen L., Liu H.-L. (2023). EPC-DARTS: Efficient partial channel connection for differentiable architecture search. Neural Netw..

[28] Zhu X., Li J., Liu Y., Wang W. (2023). Improving Differentiable Architecture Search via Self-Distillation. arXiv.

[29] Mihaljević B., Bielza C., Larrañaga P. (2021). Bayesian networks for interpretable machine learning and optimization. Neurocomputing.

[30] Karathanasopoulos N., Angelikopoulos P., Papadimitriou C., Koumoutsakos P. (2017). Bayesian identification of the tendon fascicle’s structural composition using finite element models for helical geometries. Comput. Methods Appl. Mech. Eng..

[31] Chen J., Chen M., Wen J., He L., Liu X. (2022). A Heuristic Construction Neural Network Method for the Time-Dependent Agile Earth Observation Satellite Scheduling Problem. Mathematics.

[32] Ma Z., Yuan X., Han S., Sun D., Ma Y. (2019). Improved chaotic particle swarm optimization algorithm with more symmetric distribution for numerical function optimization. Symmetry.

[33] Gharehchopogh F.S., Maleki I., Dizaji Z.A. (2022). Chaotic vortex search algorithm: Metaheuristic algorithm for feature selection. Evol. Intell..

[34] Behera M., Sarangi A., Mishra D., Mallick P.K., Shafi J., Srinivasu P.N., Ijaz M.F. (2022). Automatic Data Clustering by Hybrid Enhanced Firefly and Particle Swarm Optimization Algorithms. Mathematics.

[35] Wolpert D.H., Macready W.G. (1997). No free lunch theorems for optimization. IEEE Trans. Evol. Comput..

[36] Ang K.M., El-kenawy E.-S.M., Abdelhamid A.A., Ibrahim A., Alharbi A.H., Khafaga D.S., Tiang S.S., Lim W.H. (2022). Optimal Design of Convolutional Neural Network Architectures Using Teaching–Learning-Based Optimization for Image Classification. Symmetry.

[37] Rao R.V., Savsani V.J., Vakharia D.P. (2011). Teaching–learning-based optimization: A novel method for constrained mechanical design optimization problems. Comput.-Aided Des..

[38] Ang K.M., Lim W.H., Tiang S.S., Ang C.K., Natarajan E., Ahamed Khan M. Optimal Training of Feedforward Neural Networks Using Teaching-Learning-Based Optimization with Modified Learning Phases. Proceedings of the 12th National Technical Seminar on Unmanned System Technology 2020.

[39] Schaffer J.D., Caruana R.A., Eshelman L.J. (1990). Using genetic search to exploit the emergent behavior of neural networks. Phys. D Nonlinear Phenom..

[40] Kitano H. (1990). Empirical studies on the speed of convergence of neural network training using genetic algorithms. Proceedings of the AAAI.

[41] Stanley K.O., Miikkulainen R. (2002). Evolving neural networks through augmenting topologies. Evol. Comput..

[42] Siebel N.T., Sommer G. (2007). Evolutionary reinforcement learning of artificial neural networks. Int. J. Hybrid Intell. Syst..

[43] Stanley K.O., D’Ambrosio D.B., Gauci J. (2009). A hypercube-based encoding for evolving large-scale neural networks. Artif. Life.

[44] Banharnsakun A. (2019). Towards improving the convolutional neural networks for deep learning using the distributed artificial bee colony method. Int. J. Mach. Learn. Cybern..

[45] Zhu W., Yeh W., Chen J., Chen D., Li A., Lin Y. Evolutionary convolutional neural networks using abc. Proceedings of the 2019 11th International Conference on Machine Learning and Computing.

[46] Ozcan T., Basturk A. (2019). Transfer learning-based convolutional neural networks with heuristic optimization for hand gesture recognition. Neural Comput. Appl..

[47] Dixit U., Mishra A., Shukla A., Tiwari R. (2019). Texture classification using convolutional neural network optimized with whale optimization algorithm. SN Appl. Sci..

[48] Kylberg G. (2011). Kylberg Texture Dataset v. 1.0.

[49] Brodatz P. (1966). Textures: A Photographic Album for Artists and Designers.

[50] Ojala T., Maenpaa T., Pietikainen M., Viertola J., Kyllonen J., Huovinen S. Outex-new framework for empirical evaluation of texture analysis algorithms. Proceedings of the 2002 International Conference on Pattern Recognition.

[51] Ratre A. (2020). Stochastic gradient descent–whale optimization algorithm-based deep convolutional neural network to crowd emotion understanding. Comput. J..

[52] Murugan R., Goel T., Mirjalili S., Chakrabartty D.K. (2021). WOANet: Whale optimized deep neural network for the classification of COVID-19 from radiography images. Biocybern. Biomed. Eng..

[53] Wen L., Gao L., Li X., Li H. (2022). A new genetic algorithm based evolutionary neural architecture search for image classification. Swarm Evol. Comput..

[54] Xue Y., Wang Y., Liang J., Slowik A. (2021). A self-adaptive mutation neural architecture search algorithm based on blocks. IEEE Comput. Intell. Mag..

[55] He C., Tan H., Huang S., Cheng R. (2021). Efficient evolutionary neural architecture search by modular inheritable crossover. Swarm Evol. Comput..

[56] Xu Y., Ma Y. (2023). Evolutionary neural architecture search combining multi-branch ConvNet and improved transformer. Sci. Rep..

[57] Salih S.Q. (2019). A new training method based on black hole algorithm for convolutional neural network. J. Southwest Jiaotong Univ..

[58] Llorella F.R., Azorín J.M., Patow G. (2023). Black hole algorithm with convolutional neural networks for the creation of brain-computer interface based in visual perception and visual imagery. Neural Comput. Appl..

[59] Nguyen T., Nguyen G., Nguyen B.M. (2020). EO-CNN: An enhanced CNN model trained by equilibrium optimization for traffic transportation prediction. Procedia Comput. Sci..

[60] Nandhini S., Ashokkumar K. (2022). An automatic plant leaf disease identification using DenseNet-121 architecture with a mutation-based henry gas solubility optimization algorithm. Neural Comput. Appl..

[61] Pandey A., Jain K. (2022). Plant leaf disease classification using deep attention residual network optimized by opposition-based symbiotic organisms search algorithm. Neural Comput. Appl..

[62] Junior F.E.F., Yen G.G. (2019). Particle swarm optimization of deep neural networks architectures for image classification. Swarm Evol. Comput..

[63] Zeiler M.D. (2012). Adadelta: An adaptive learning rate method. arXiv.

[64] Glorot X., Bengio Y. Understanding the difficulty of training deep feedforward neural networks. Proceedings of the Thirteenth International Conference on Artificial Intelligence and Statistics.

[65] Lydia A., Francis S. (2019). Adagrad—An optimizer for stochastic gradient descent. Int. J. Inf. Comput. Sci..

[66] Kingma D.P., Ba J. (2014). Adam: A method for stochastic optimization. arXiv.

[67] He K., Zhang X., Ren S., Sun J. Delving deep into rectifiers: Surpassing human-level performance on imagenet classification. Proceedings of the IEEE International Conference on Computer Vision.

[68] Zeng X., Zhang Z., Wang D. AdaMax Online Training for Speech Recognition. http://cslt.riit.tsinghua.edu.cn/mediawiki/images/d/df/Adamax_Online_Training_for_Speech_Recognition.pdf..

[69] Ruder S. (2016). An overview of gradient descent optimization algorithms. arXiv.

[70] LeCun Y., Bottou L., Bengio Y., Haffner P. (1998). Gradient-based learning applied to document recognition. Proc. IEEE.

[71] Larochelle H., Erhan D., Courville A., Bergstra J., Bengio Y. An empirical evaluation of deep architectures on problems with many factors of variation. Proceedings of the 24th International Conference on Machine Learning.

[72] Xiao H., Rasul K., Vollgraf R. (2017). Fashion-mnist: A novel image dataset for benchmarking machine learning algorithms. arXiv.

[73] Bruna J., Mallat S. (2013). Invariant scattering convolution networks. IEEE Trans. Pattern Anal. Mach. Intell..

[74] Chan T.-H., Jia K., Gao S., Lu J., Zeng Z., Ma Y. (2015). PCANet: A simple deep learning baseline for image classification?. IEEE Trans. Image Process..

[75] Rifai S., Vincent P., Muller X., Glorot X., Bengio Y. Contractive auto-encoders: Explicit invariance during feature extraction. Proceedings of the International Conference on Machine Learning.

[76] Sun Y., Xue B., Zhang M., Yen G.G. (2019). Evolving deep convolutional neural networks for image classification. IEEE Trans. Evol. Comput..

[77] Krizhevsky A., Sutskever I., Hinton G.E. (2017). Imagenet classification with deep convolutional neural networks. Commun. ACM.

[78] Iandola F.N., Han S., Moskewicz M.W., Ashraf K., Dally W.J., Keutzer K. (2016). SqueezeNet: AlexNet-level accuracy with 50x fewer parameters and <0.5 MB model size. arXiv.

[79] Derrac J., García S., Molina D., Herrera F. (2011). A practical tutorial on the use of nonparametric statistical tests as a methodology for comparing evolutionary and swarm intelligence algorithms. Swarm Evol. Comput..

[80] Springenberg J.T., Dosovitskiy A., Brox T., Riedmiller M. (2014). Striving for simplicity: The all convolutional net. arXiv.

